# GeNetOntology: identifying affected gene ontology terms via grouping, scoring, and modeling of gene expression data utilizing biological knowledge-based machine learning

**DOI:** 10.3389/fgene.2023.1139082

**Published:** 2023-08-21

**Authors:** Nur Sebnem Ersoz, Burcu Bakir-Gungor, Malik Yousef

**Affiliations:** ^1^ Department of Bioengineering, Graduate School of Engineering and Science, Abdullah Gul University, Kayseri, Türkiye; ^2^ Department of Computer Engineering, Faculty of Engineering, Abdullah Gul University, Kayseri, Türkiye; ^3^ Department of Bioengineering, Faculty of Life and Natural Sciences, Abdullah Gul University, Kayseri, Türkiye; ^4^ Department of Information Systems, Zefat Academic College, Zefat, Israel; ^5^ Galilee Digital Health Research Center (GDH), Zefat Academic College, Zefat, Israel

**Keywords:** gene ontology, gene expression data analysis, machine learning, feature selection, enrichment analysis, feature scoring, feature grouping, classification

## Abstract

**Introduction:** Identifying significant sets of genes that are up/downregulated under specific conditions is vital to understand disease development mechanisms at the molecular level. Along this line, in order to analyze transcriptomic data, several computational feature selection (i.e., gene selection) methods have been proposed. On the other hand, uncovering the core functions of the selected genes provides a deep understanding of diseases. In order to address this problem, biological domain knowledge-based feature selection methods have been proposed. Unlike computational gene selection approaches, these domain knowledge-based methods take the underlying biology into account and integrate knowledge from external biological resources. Gene Ontology (GO) is one such biological resource that provides ontology terms for defining the molecular function, cellular component, and biological process of the gene product.

**Methods:** In this study, we developed a tool named GeNetOntology which performs GO-based feature selection for gene expression data analysis. In the proposed approach, the process of Grouping, Scoring, and Modeling (G-S-M) is used to identify significant GO terms. GO information has been used as the grouping information, which has been embedded into a machine learning (ML) algorithm to select informative ontology terms. The genes annotated with the selected ontology terms have been used in the training part to carry out the classification task of the ML model. The output is an important set of ontologies for the two-class classification task applied to gene expression data for a given phenotype.

**Results:** Our approach has been tested on 11 different gene expression datasets, and the results showed that GeNetOntology successfully identified important disease-related ontology terms to be used in the classification model.

**Discussion:** GeNetOntology will assist geneticists and scientists to identify a range of disease-related genes and ontologies in transcriptomic data analysis, and it will also help doctors design diagnosis platforms and improve patient treatment plans.

## 1 Introduction

Precision medicine gained importance in the last decade (König et al., 2017). Molecular abnormalities in disease formation can differ from patient to patient, and a more customized medication plan is required for each individual. Fortunately, today’s next-generation sequencing technologies offer several opportunities to quickly generate a series of omics data in order to monitor molecular alterations at different levels. Along this line, next-generation sequencing has been widely used to analyze genetic variations, gene-expression profiling, epigenomes, and genome diversity (Levy and Boone, 2019). Furthermore, with improvement in the technology, the cost of these high-throughput technologies is decreasing. However, the reduction in the cost of next-generation sequencing and other high-throughput technologies creates a burden on the data analysis approaches.

Biological systems are quite complex by their nature. Therefore, one of the difficulties in omics data analysis stems from these complex data, where the information is present at multiple layers. For example, in a biological system, the gene activities under different physiological states are reflected by the gene expression data (at the transcriptome level). On the other hand, the epigenome reflects the effects of environmental factors on gene activities and disease development. Epigenetic modifications such as DNA methylations and histone modifications (acetylation, methylation, phosphorylation, and ubiquitination) can alter the genome and regulate gene expression. Hence, epigenomic variations can control transcriptomes which can cause proteomic dysfunctionalities and result in disease formation. A similar scenario may also be caused by genomic variations. In addition to that, post-translational modifications play roles in phenotypic differentiation in physiology and pathology. Such complexities of biological systems make it harder to analyze biological data.

In order to enlighten the molecular and functional mechanisms of disease development, one of the widely studied data types is transcriptomic data (Barrett et al., 2012; Perscheid et al., 2019). Researchers analyze gene expression datasets to identify disease-associated genes and to find biomarkers that can aid in early diagnosis and targeted therapies. Various technologies, such as microarray and RNA-seq technology, can detect thousands of transcripts. One of the challenges in gene expression data analysis stems from it being noisy and high dimensional. It has a high number of features (i.e., genes or mRNAs) measured as a result of the experiments conducted at the molecular level, with a small number of samples including the patient group, control group, and treated or non-treated groups. The expression values of the genes are provided to a learning algorithm to accomplish the classification task. However, usually, the majority of the genes (i.e., features) are redundant, noisy, or irrelevant to the learning task, which will influence the learning accuracy and training speed (Ang et al., 2016; Aziz et al., 2017). In other words, only a number of genes are primarily related to disease development. Therefore, it is crucial to find disease-related transcripts (i.e., genes) by applying feature selection methods. In other words, gene selection refers to biomarker detection via applying feature selection methods on gene expression data (Perscheid, 2021).

Traditional feature selection (FS) approaches are mainly based on statistical tests. In the literature, several comparative studies were carried out on different FS methods (Albattah et al., 2022). Several comprehensive studies of different FS methods are provided for classification problems (Bolón-Canedo et al., 2016). Yet, another recent survey discussed the basics, applications, and challenges of FS methods in the context of high-dimensional data (Abdulwahab et al., 2022). According to the interaction of the FS method with the classification model, Liu and Motoda (1998) originally classified the FS methods into filter, wrapper, and embedded methods (Guyon et al., 2006; Jensen and Shen, 2008; Chandrashekar and Sahin, 2014; You et al., 2014; Tadist et al., 2019; Albattah et al., 2022). Later on, hybrid and ensemble methods were proposed in the literature as variants of them (Bellazzi and Zupan, 2007; Ang et al., 2016; Perscheid et al., 2019; Tadist et al., 2019).

Filter methods evaluate a subset of features or a feature only by using the intrinsic properties of the training samples. These methods can be combined with a variety of classifiers, and therefore, filter methods have a better generalization ability and lower computational complexity. Filter methods are based on F-statistics (ANOVA, t-test, etc.), mutual information, and entropy (Srinivasa et al., 2020), and they evaluate the influence of the input values on the output value. Some examples of the filter methods are information gain (IG) and ReliefF (Perscheid et al., 2019).

Wrapper methods can achieve better classification performance than filter methods because they are specific to a particular classifier (Inza et al., 2004). These methods assess the quality of a candidate subset. The successive feature selection (SFS) approach is an example of the wrapper-type feature selection method (Perscheid et al., 2019). The main disadvantage of the wrapper method is that they are far more time-consuming.

As a special case of the wrapper methods, embedded methods are characterized by an interaction between the FS and the classification algorithm. When embedded methods are used to construct the classifier, feature subsets are generated (Wang et al., 2015). The support vector machine with recursive feature elimination (SVM-RFE) approach is an example of embedded FS methods. As summarized here, traditional methods are fully data-driven approaches, and they neglect biological domain knowledge. For example, when selecting important genes in transcriptome data analysis, the importance of each gene is usually evaluated with a filtering method, ignoring the interactions and relationships between the genes. On the other hand, wrapper methods utilize the learning algorithm while evaluating the features (i.e., genes). Hence, they are able to find optimal feature sets, but they may encounter the overfitting problem.

It is reported that one of the main obstacles of traditional methodologies is that they hardly perform biological interpretation, and hence they do not allow the creation of new biological knowledge (Perscheid et al., 2019; Yousef et al., 2021). Since traditional gene selection approaches have limitations like noise due to the high-dimensional data (Perscheid, 2021), lately, scientists have started to develop integrative gene selection approaches that incorporate domain knowledge from external biological resources during the gene expression data analysis (Perscheid et al., 2019; Yousef et al., 2021). Genes perform their biological functions in an organized fashion (in terms of metabolic networks and signaling pathways). Hence, scientists attempt to develop new methodologies which employ external biological information such as pathways, interactions, and gene ontology (Yousef et al., 2021). To this end, the integrative gene selection process generates a ranked list of genes according to both statistical metrics and biological background information collected from external resources (Perscheid et al., 2019).

There are several resources, databases, and repositories of biological knowledge. For example, Kyoto Encyclopedia of Genes and Genomes (KEGG) is a widely used external ontology resource, which serves as a pathway knowledge-base for systemic analysis of gene functions, and it provides manually curated pathways (Kanehisa and Goto, 2000). As another resource, The Cancer Genome Atlas (TCGA) (Tomczak et al., 2015) hosts detailed information about oncogenomic expression profiles. On the other hand, miRTarbase (Chou et al., 2018) serves annotated experimentally validated miRNA–target interactions. An integrative gene selection method could also utilize functional information from the UniProt Knowledge Base (UniProtKB) (The UniProt Consortium, 2017). DisGeNET provides a system-level view on the genes and diseases via giving access to other data sources such as RNA and interaction graphs (Perscheid, 2021). As one of the widely used biological knowledge bases, GO intends to unite detailed and standardized terminologies defined for the various levels of molecular biology (Balakrishnan et al., 2013). It supplies tools for exploring these terminologies and for describing biological terms using this vocabulary (The Gene Ontology Consortium, 2019).

The GO Consortium created GO with the aim of presenting a fully defined, organized terminology to describe the gene functions and products in each organism (Ashburner et al., 2000). Primarily, Mouse Genome Informatics (MGI), FlyBase, and *Saccharomyces* Genome Database (SGD) model organism databases were used by the GO Consortium and then were expanded to many organisms. In the ontology, over 45,000 terms have been connected by about 134,000 relations. In addition, more than 7 million genes and the annotations of gene products from over 3,200 organisms are included in the GO knowledge base (The Gene Ontology Consortium, 2019). Three different aspects of genes are covered by GO, i.e., biological process (BP), molecular function (MF), and cellular component (CC). Although MF represents the activity of a gene product at the molecular level, CC represents the cellular localization of the gene product, or where it acts. Lastly, the BP is the larger biological objective that the molecular-level process of the gene product contributes to The Gene Ontology Consortium (2019).

GO annotations constructed by connecting specific gene products to terms in the ontology are also contained in the GO knowledge base. Each information includes evidence on which it is based, using the standardized codes, computational analysis evidence codes such as Inferred from Sequence or structural Similarity (ISS), curatorial statement evidence codes such as Inferred by Curator (IC), and electronic annotation evidence code such as inferred from electronic annotation (IEA), where these codes are defined by the Evidence and Conclusion Ontology (ECO) (Chibucos et al., 2017; Guide to GO evidence codes, 2022).

Recently, Perscheid published a survey on prior knowledge-based approaches for integrative biomarker detection from gene expression datasets (Perscheid, 2021). In that article, she evaluated the respective characteristics of different integrative gene selection approaches and presented an overview of external knowledge bases that are utilized in these approaches (Perscheid, 2021). The article reported that GO and KEGG resources are predominantly used as external knowledge bases for integrative gene selection. For example, Qi and Tang (2007) showed that incorporating GO as a biological knowledge outperforms traditional gene selection methods in microarray data analysis. Another approach used GO and KEGG ontologies to filter genes more accurately (Fang et al., 2014). GO terms are also used in another study to show the limitations of network-based annotations (Asif et al., 2018).

The same review paper (Perscheid, 2021) noted that although prior knowledge-based approaches offer several advantages for gene selection, these approaches require advanced integration concepts to consider both statistical and biological characteristics. As a result, these approaches have not been widely adopted. In this respect, recently, we proposed a Grouping–Scoring–Modeling (G-S-M) approach (Yousef et al., 2021) for integrating biological knowledge into the machine learning model. The G-S-M approach selects a set of features where different sets can be generated via 1) using pre-existing biological knowledge stored in a database (such as mirTarBase (Chou et al., 2018), DisGeNET (Piñero et al., 2015), and KEGG pathways (Kanehisa and Goto, 2000)) or 2) fully data-driven approach using statistical measures such as Pearson’s correlations. The G-S-M approach has been utilized in the development of different computational tools. Examples of such tools are maTE (Yousef et al., 2019) that uses microRNA target gene information for grouping the genes; miRcorrNet (Yousef et al., 2021) and miRModuleNet (Yousef et al., 2022), which detect feature sets via concurrently analyzing mRNA and miRNA expression datasets, respectively; CogNet (Yousef et al., 2021) and PriPath (Yousef et al., 2022) that use KEGG pathway information for grouping the genes; GediNet (Qumsiyeh et al., 2022) that uses disease gene associations from DisGeNET while defining the sets of the genes; and miRdisNET (Jabeer et al., 2023) that uses miRNA target gene information while assigning the genes into sets. As the recent review paper (Perscheid, 2021) points out, biomarker detection only based on statistical analysis is insufficient. To this end, here, we attempt to incorporate external biological knowledge into the selection process, and hence we aim to deliver biologically relevant results. In other words, this study mainly focuses on the detection of disease signatures and on the discovery of novel gene sets with relations across a subset of GO terms for the disease under investigation. In this study, our main aim is to assign genes into groups using the G-S-M approach and to identify the highly correlated sets of GO terms that are related to the disease under investigation. Along this line, in this study, the GeNetOntology algorithm is proposed as a novel algorithm that improves classification performance by utilizing GO as external biological information while selecting the most relevant genes from gene expression datasets. In our experiments, the Monte Carlo cross-validation (MCCV) technique is utilized. Hence, in each iteration, some samples are selected randomly for the training set, and the rest of the samples are selected for the testing set. In each training iteration, the most informative GO term is identified. Later, the genes that are associated with the top-ranked GO term in each iteration is merged to train the model. In addition, we perform comparative evaluation with other existing methods. The novelty and originality of our approach stems from its capability to explore GO terms to classify and to find the most relevant sets of GO terms associated with the disease under study. In this respect, our approach differs from traditional gene selection approaches where searching is carried out by considering individual genes.

## 2 Materials and methods

### 2.1 Gene expression dataset

A total of 11 gene expression datasets for different types of human complex diseases were downloaded from Gene Expression Omnibus (GEO) (https://www.ncbi.nlm.nih.gov/geo/). All datasets include both healthy samples (labeled as negative) and patient samples (labeled as positive). These datasets are used to test the performance of GeNetOntology and to compare it with that of other tools. The gene expression dataset is represented as a matrix. In this matrix, while genes (i.e., mRNAs) are shown in the columns, rows represent the samples. This matrix contains a special column called label, which indicates the class annotation for each row. Here, the class labels are either positive, indicating the patient, or negative, indicating the control. Table 1 presents the GEO accession numbers, titles, PubMed Identification numbers (PMID), disease name, and numbers of cases and controls for each one of the 11 gene expression datasets.

**TABLE 1:** Description of the 11 gene expression datasets that have been used in this study. Each entry has the GEO Accession, PMID, disease type, number of healthy samples (controls), and number of patients.

Title	GEO accession	PMID		Disease type	# Of healthy	# Of patients
Glioma-derived stem cell factor effect on angiogenesis in the brain	GDS1962	16616334	Glioma		23	157
Early-stage Parkinson’s disease	GDS2519	17215369	Parkinson’s disease		23	50
		18669654				
Metastatic prostate cancer (HG-U95C)	GDS2545	17430594	Prostate cancer		81	90
		15254046				
Metastatic prostate cancer (HG-U95C)	GDS2547	17430594	Prostate cancer		75	89
		15254046				
Large airway epithelial cells from cigarette smokers with suspected lung cancer	GDS2771	17334370	Lung cancer		90	102
		20375364				
Cigarette smoking effect on lung adenocarcinoma	GDS3257	18297132	Lung adenocarcinoma		49	58
Colon epithelial biopsies of ulcerative colitis patients	GDS3268	18523026	Colitis		73	129
Non-small cell lung carcinoma in female non-smokers	GDS3837	20802022	Lung cancer		60	60
		25889623				
Pediatric acute leukemia patients with early relapse: white blood cells	GDS4206	21295523	Leukemia		157	40
Colorectal cancer: laser microdissected tumor tissues	GDS4516_4718	21270110	Colorectal cancer		44	104
Pulmonary hypertension: PBMCs	GDS5499	22545094	Pulmonary hypertension		41	99

### 2.2 Gene ontology data

The GO (‘Gene Ontology Consortium: going forward’, 2015) (http://www. geneontology.org) database provides the biological knowledge that will be used for the grouping component. The GO and Human Phenotype Ontology (HPO) data are downloaded from the Molecular Signatures Database (*GSEA | MSigDB | Browse Human Gene Setsgui*) (Liberzon et al., 2015). HPO terms and all GO terms from GO BP, GO CC, and GO MF categories are used in this study. The numbers of the GO terms in each category and the number of HPO terms are listed in Table 2. As illustrated in Supplementary Figure S1, each GO term is associated with one or more genes and it can be represented as a gene set.

**TABLE 2:** Summary of the GO subsets with the number of terms associated with each GO subset.

Subset of gene ontology (GO)	#Ontology groups (terms)
All ontology gene sets	14,998
GO biological process (BP)	7,481
GO molecular functions (MF)	1,708
GO cellular component (CC)	996
HPO	4,813

We wanted to see the distribution of the number of genes that are associated with each GO term. For this purpose, for each one of the BP, CC, and MF categories, we further divide GO terms into bins based on the number of genes that are associated with each GO term. Supplementary Figure S2 presents three histograms for each one of the GO BP, CC, and MF categories. In each histogram, we plot the counts of GO terms, where n genes are associated with that GO term and n increases in a window of 20. When the number of genes associated with a GO term (n) is increased by 20, we get 7,481 bins for the BP category, 996 bins for the CC category, and 1,708 bins for the MF category.

Genes that are annotated with the same GO term either share a common function, perform similar activity depending on their responsibility at the molecular or cellular level, or act within the same cellular component. Genes that are annotated with GO Biological Process play roles in biological processes. Furthermore, each gene that is annotated with a specific GO biological process term performs a specific biological process. For example, genes that are annotated with the GOBP_Artery_Morphogenesis term are responsible for artery morphogenesis.

### 2.3 The general G-S-M model

The main idea of the G-S-M technique is to perform the scoring operation for different sets of features rather than selecting and evaluating individual features. Biological knowledge is used as a function that is applied on the feature space to create sets of features, where each set includes one or more features, i.e., one or more genes in the gene selection problem.

The general G-S-M technique was developed by Yousef et al. (2019) and was embedded in different computational tools, such as SVM-RCE-R (Yousef et al., 2020), miRcorrNet (Yousef et al., 2021), maTE (Yousef et al., 2019), CogNet (Yousef et al., 2021), SVM-RCE-R-OPT (Yousef et al., 2021), Integrating GO-based Grouping and Ranking (Yousef et al., 2021), PriPath (Yousef et al., 2023), miRdisNET (Jabeer et al., 2023), GediNET (Qumsiyeh et al., 2022), miRModuleNet (Yousef et al., 2022), AMP-GSM (Söylemez et al., 2023), and TextNetTopics (Yousef and Voskergian, 2022). The main idea and most of the relevant tools are reviewed in Yousef et al. (2021).

#### 2.3.1 General methodology of GeNetOntology

**FIGURE 1:**
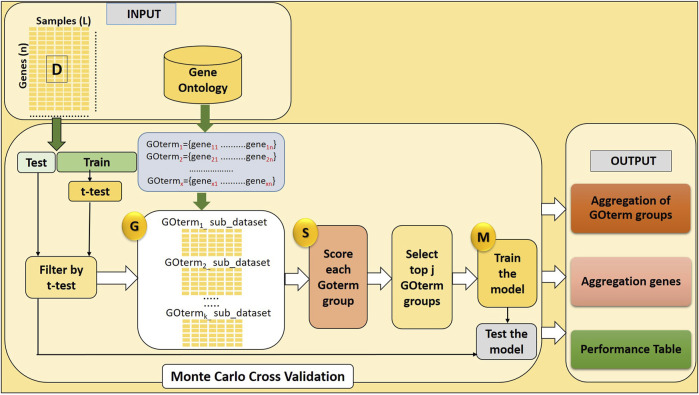
GeNetOntology consists of four major components: GO term groups are extracted; sub-datasets for each GO term groups are generated by G component; component S scores and ranks the groups; and component M creates and evaluates the model.

Here, we develop a novel approach named GeNetOntology. The GeNetOntology consists of three main components (illustrated in Figure 1):

1. G Component: to generate sub-datasets for each GO term group.
2. S Component: to score GO terms.
3. M Component: to train the classifier (Random Forest) to build the model.

The main purpose of the GeNetOntology is to find significant GO terms (scored in S Component) to be used for training the classifier (M component). In order to evaluate a set of features, for each GO term, a sub-dataset is created by only including the expression values of the genes which are associated with that particular GO term. The pseudo-code of GeNetOntology is presented in Supplementary Table S1, and the algorithm is explained more in detail in the following sections.

The gene expression dataset is represented by C, which consists of two parts, C_train_ and C_test_. Although C_train_ has been utilized for scoring the GO terms and training the classifier to create a model, C_test_ has been used to test and report the final performance.

#### 2.3.2 Component G: generating sub-datasets

**FIGURE 2:**
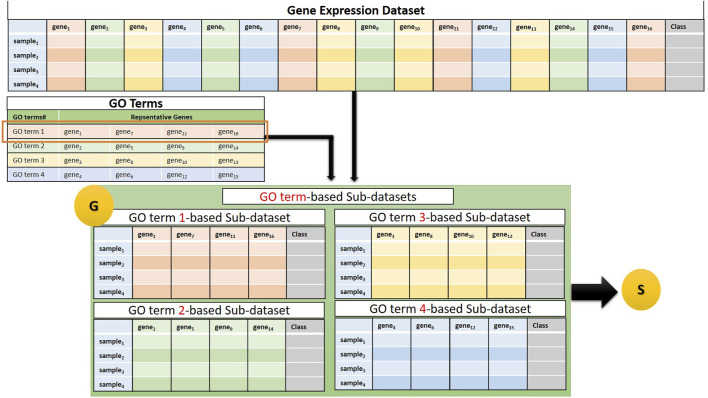
Example of a sub-dataset generation based on genes that belong to a GO term. The generated sub-datasets are then subject to the S, the Scoring Component.

The G component of GeNetOntology creates sub-datasets for each GO term. Several genes are annotated with a specific GO term. For each GO term, the G component extracts a sub-dataset from the gene expression dataset. In other words, each sub-dataset includes 1) expression values just for the genes that are annotated with that particular GO term and 2) class labels (positive or negative) of the samples. We would like to note that each sub-dataset includes the same number of samples, but different numbers of features depending on the number of genes that are annotated with that particular GO term. Figure 2 presents a flowchart for sub-dataset generation based on the genes that are annotated with a specific GO term. Each sub-dataset is named as GO_term(i)_sub_dataset_, where i starts from 1 and increases until k. Here, k refers to the number of GO terms exported.

The G component involves two tables:

(1) The GO Terms table: Each GO term is associated with a set of genes (shown as a row of the GO Terms table in Figure 2).
(2) Gene expression dataset: The flowchart in Figure 2 illustrates the generation of four sub-datasets, each corresponding to a specific GO term. Next, component S, which is shown as “S” in the last part of Figure 2, performs the scoring step by taking these sub-datasets as an input.

#### 2.3.3 Component S: scoring GO terms

S, as a second component, takes the created sub-datasets as an input from component G and operates an ML algorithm (Random Forest) with an internal MCCV repeated r times (as shown in Supplementary Figure S3). It has been performed on each sub-dataset to give a score for its related GO term. The scoring is evaluated by testing the capability of each GO term in terms of its classification performance. In other words, the score implies the accuracy of the classification by only using the gene expression values of the genes that are annotated with that specific GO term. In the S component, the classification accuracy is averaged over the r iterations of the MCCV. The mean accuracy value is used as the final score of the particular GO term. The S component ranks all GO terms according to their scores. The top-scoring GO terms are used in the next component to train the model.

Table 3 presents an example of the S output where for each GO term, we assign a score that is calculated as the mean accuracy.

**TABLE 3:** Example of grouping Gene Ontology (GO) terms and their computed scores. Calculated for the GO BP category in the GDS1962 dataset.

GO term#	Score
GOBP_AMEBOIDAL_TYPE_CELL_MIGRATION	1
GOBP_APOPTOTIC_PROCESS_INVOLVED_IN_BLOOD_VESSEL_MORPHOGENESIS	1
GOBP_CANONICAL_WNT_SIGNALING_PATHWAY	0.98
GOBP_CELLULAR_RESPONSE_TO_EXTERNAL_STIMULUS	0.98
GOBP_CELLULAR_RESPONSE_TO_LOW_DENSITY_LIPOPROTEIN_PARTICLE_STIMULUS	0.97
GOBP_CELLULAR_RESPONSE_TO_NUTRIENT	0.96
GOBP_CELLULAR_RESPONSE_TO_PEPTIDE_HORMONE_STIMULUS	0.96
GOBP_CHOLESTEROL_STORAGE	0.95
GOBP_FATTY_ACID_BIOSYNTHETIC_PROCESS	0.93
GOBP_FOAM_CELL_DIFFERENTIATION	0.92

#### 2.3.4 Component M: building the model

The M component trains the classifier and creates the model. The main principle of Component M is illustrated in Supplementary Figure S4. This component trains a classifier (a Random Forest model) using the gene expression values of the genes that are annotated with the top-scoring GO terms. We repeat this procedure in a cumulative manner. In the first iteration, a Random Forest model is built by operating only on the genes that are annotated with the highest scoring GO term. In the second iteration, the M component takes the second highest scoring GO term and merges the genes annotated with this GO term with the gene set annotated with the highest scoring GO term which is identified in the first iteration. In this way, it forms a new sub-dataset that will be subject to training and testing the model. This operation continues in the same way until all GO terms are processed. Hence, we calculate the cumulative performance of the model. Through this approach, it becomes possible to plot the performance results over different feature sets (i.e., the highest scoring GO term, the top two highest scoring GO terms, until the top 10 highest scoring GO terms). In this way, one can discover the best feature set which is defined as the combination of the genes annotated with the top scoring GO terms.

A sample output of the M component is shown in Table 4. GeNetOntology presents the average performance metrics of the classification using the genes that are associated with the top 10 scoring GO terms, averaged over 10-fold MCCV.

**TABLE 4:** Sample output of GeNetOntology. Averages over 10-fold MCCV are presented for different performance metrics. Obtained cumulatively for the top 10ranked Gene Ontology BP groups using the GDS1962 dataset.

#Groups	#Genes	Accuracy	Sensitivity	Specificity	AUC
10	133.9	0.94	0.96	0.9	1
9	122.9	0.95	0.96	0.95	0.995
8	114.9	0.94	0.96	0.9	0.985
7	107.8	0.95	0.96	0.95	0.98
6	93.8	0.94	0.98	0.85	0.99
5	88.6	0.95	0.98	0.9	1
4	74.7	0.98	0.98	1	1
3	62.4	0.95	0.96	0.95	1
2	48.2	0.94	0.98	0.85	0.98
1	31.9	0.91	0.96	0.8	0.97

### 2.4 Design and implementation of GeNetOntology

Figure 1 and Supplementary Figure S5 demonstrate the workflow of GeNetOntology. Two input files are required by GeNetOntology: gene expression data and the biological knowledge file (GO terms and a set of genes annotated with each GO term). In this study, GO terms are used for grouping the genes and for grouping their associated gene expression values. In this way, we generate different sets of features using biological knowledge, and then we evaluate the contribution of each feature set to the classification task.

The gene expression data are separated randomly into training and testing parts. The testing data are only used to evaluate the model’s performance. The G-S-M components are followed sequentially. The whole workflow is repeated N times, where we set N to 10 in this study. This repetitive part is shown in the MCCV box in Figure 1 and in Supplementary Figure S5.

At the initial steps of GeNetOntology, in order to filter the least significant genes on the training set, a *t*-test is performed. Additionally, to be able to have the same representation of the genes in the test dataset, only the selected genes within the training set are used in the test set. The test set is only used at the M component for testing the model. The MCCV loop creates N lists of different outputs, such as lists of performance tables, lists of ranked GO terms, and ranked genes. The average is calculated over all lists of performance tables to create a final performance table that also contains the standard deviation. The robust rank aggregation approach was applied to the other lists (i.e., ranked GO terms and ranked genes) to aggregate them into a final list, as shown in Table 5. The ranked GO terms and the genes that are annotated with these terms are shown in the final list*.* All those final lists or tables are visualized in the output panel of GeNetOntology, as shown in Figure 1 and Supplementary Figure S5.

**TABLE 5:** Example of the robust rank aggregation output for the GDS1962 dataset where the ranked GO BP terms (groups) and their associated genes are shown.

GO groups	*p*-value (Score)	#Genes	Genes
GOBP_ARTERY_MORPHOGENESIS	1.30E-07	17	ADAMTS9, APOE, PRRX1, EFEMP2, VEGFA…
GOBP_CHROMATIN_SILENCING	4.44853E-07	9	SMCHD1, HMGB1, EZH2…
GOBP_CAMERA_TYPE_EYE_DEVELOPMENT	5.61732E-07	49	RDH10, MEGF11, ATOH7…
GOBP_GLAND_DEVELOPMENT	7.07998E-07	85	PRMT5, IQGAP3, MSN…
GOBP_RESPONSE_TO_HORMONE	1.65122E-06	123	IDH1, ADAM9, GPR173…
GOBP_NEGATIVE_REGULATION_OF_EPITHELIAL_CELL_MIGRATION	1.8769E-06	14	HMGB1, SP100, APOE…

GeNetOntology has been implemented in the free and open-source KNIME Analytics Platform, which is a data analysis, reporting, and integration tool under the General Public License (GNU) (Berthold et al., 2009). KNIME is able to utilize scripts in both R and Python. In the KNIME workflow, there are several nodes with their own functions. Nodes have been collected under meta-nodes that fulfill a specific task. The GeNetOntology KNIME workflow is available publicly in https://github.com/malikyousef/GeNetOntology.git.

### 2.5 Model performance evaluation of GeNetOntology

We have evaluated a set of statistical measures such as specificity, sensitivity, and accuracy for each model to score model efficiency. The following formulations were used to calculate the statistics:

• Sensitivity (Recall) = True Positive/(True Positive + False Negative)
• Specificity = True Negative/(True Negative + False Positive)
• Accuracy = (True Positive + True Negative)/#All examples,

In addition, the area under the receiver operating characteristic (ROC) curve (AUC) is used to approximate the probability of a classifier which would score a randomly selected positive instance higher than a randomly selected negative instance. All reported performance measures indicate the average of 10-fold MCCV. We have performed an under-sampling approach to deal with the imbalanced dataset problem. This approach decreases the number of samples in the majority class to the number of samples in the minority class. In that way, we can reduce the bias in the size distribution of datasets and overcome the imbalanced class distribution problem. The under-sampling ratio is chosen as 1:2. Graphs and figures have been generated using the software GraphPad Prism 8.

### 2.6 Protein–protein interaction (PPI) network analysis

Network analysis was performed using Cytoscape (Franz et al., 2016). Using Cytoscape, we have visualized the PPI networks of the genes which are annotated with the most significant GO term. Cytoscape imports the human PPI network from the STRING database. The betweenness centrality of nodes was calculated in Cytoscape using the built-in NetworkAnalyzer (Cytoscape App Store - NetworkAnalyzer, 2021). For each GO category (BP, CC, and MF), genes that are annotated with the top 10 scoring GO terms were selected. For each protein, we have computed the betweenness centrality which indicates the amount of control that this node exerts over the interactions of other nodes in the network (Yoon et al., 2006). The color and size of the node are used to represent the betweenness centrality. Bigger and darker colored nodes (proteins) in the PPI network have higher betweenness centrality.

## 3 Results

### 3.1 Model performance evaluation of GeNetOntology

GeNetOntology is tested on 11 different gene expression datasets, where the characteristics of the datasets are presented in Table 1. For each dataset, for different numbers of feature sets, the accuracy, sensitivity, specificity, and AUC values have been calculated as the mean of the values obtained in 10 iterations of the cross-validation procedure. For each feature set, GeNetOntology reports the number of features (i.e., genes) included in the set (i.e., GO term). In addition, the average gene number over 10 iterations is reported. Table 4 shows the performance metrics of GeNetOntology for the GDS1962 dataset for the top 10 scoring GO terms. For example, there are 31.9 genes on average as shown in the # of the Genes column of the last row (top scoring GO term) in Table 4. There are 48.2 genes on average as shown in the # of the Genes column of the 2nd last row (top two scoring GO terms cumulatively). In other words, the model that is generated via only using the gene expression values of 48.2 genes can successfully predict glioma patients with 0.98 AUC score.

In our experiments on various gene expression datasets, three different GO categories, i.e., BP, CC, MF, and all GO terms are utilized. Table 6 summarizes the performance metrics obtained for 11 different gene expression datasets using only the top two scoring GO terms. AUC, accuracy, and sensitivity values for GDS2519 and GDS4206 are not as high as in other datasets. However, AUC, accuracy, and sensitivity values for GDS 1962, GDS3837, GDS4516_718, and GDS5499 are quite high.

**TABLE 6:** Performance results table including accuracy, sensitivity, specificity, and AUC value of GeNetOntology for 11 different datasets.

Accession Numbers in GEO	#Genes	Accuracy	Sensitivity	Specificity	AUC
GDS1962	48.2	0.94	0.98	0.85	0.98
GDS2519	170.4	0.5	0.6	0.41	0.58
GDS2545	110.5	0.72	0.72	0.7375	0.78
GDS2547	81.7	0.75	0.725	0.7875	0.83
GDS2771	68.4	0.66	0.73	0.6	0.68
GDS3257	20.6	1	1	1	1
GDS3268	90.3	0.66	0.62	0.71	0.73
GDS3837	64.6	0.98	0.96	1	0.99
GDS4206	46.1	0.63	0.25	0.8	0.55
GDS4516_718	50.6	1	1	1	1
GDS5499	52.1	0.9	0.94	0.8	0.95

Additionally, to be able to compare our approach with other solutions, we have performed additional experiments. In this respect, we have applied different traditional feature selection methods such as eXtreme Gradient Boosting (XGB) (Athanasiou et al., 2020; Li et al., 2020), Information Gain (IG) (Lei, 2012), Select K Best (SKB) (Pedregosa et al., 2011), and Fast Correlation-Based Filter (FCBF) (Senliol et al., 2008); and different classifiers such as Adaboost, Decision Tree (DT), LogitBoost, Random Forest (RF), SVM_opt, Stack_Logitboost_Kmeans, and Stack_SVM_Kmeans on the same 11 gene expression datasets using 10-fold MCCV. For each dataset, we evaluate the performance of each classifier and each feature selection method with the same number of features used by GeNetOntology. For most of the tested classifiers, the AUC of the XGB FS method (Supplementary Table S2) showed higher performance than the IG FS method (Supplementary Table S3), SKB (Supplementary Table S4), and FCBF FS method (Supplementary Table S5). For each FS method, we have plotted the average AUC values over seven different classifiers for 11 different datasets. As shown in Supplementary Figure S6, GeNetOntology generates similar AUC values compared with other methods, averaged over different datasets. We would like to emphasize that the aim of GeNetOntology is not to compete with other feature selection (FS) approaches. Our aim is to select significant ontology terms that have biological meaning. Even if the performance of GeNetOntology is similar with that of other FS methods or even slightly less than that of other FS methods, the contribution of the tool is to find the most informative GO terms that can help the researchers understand the biological background of the disease under study.

**FIGURE 3:**
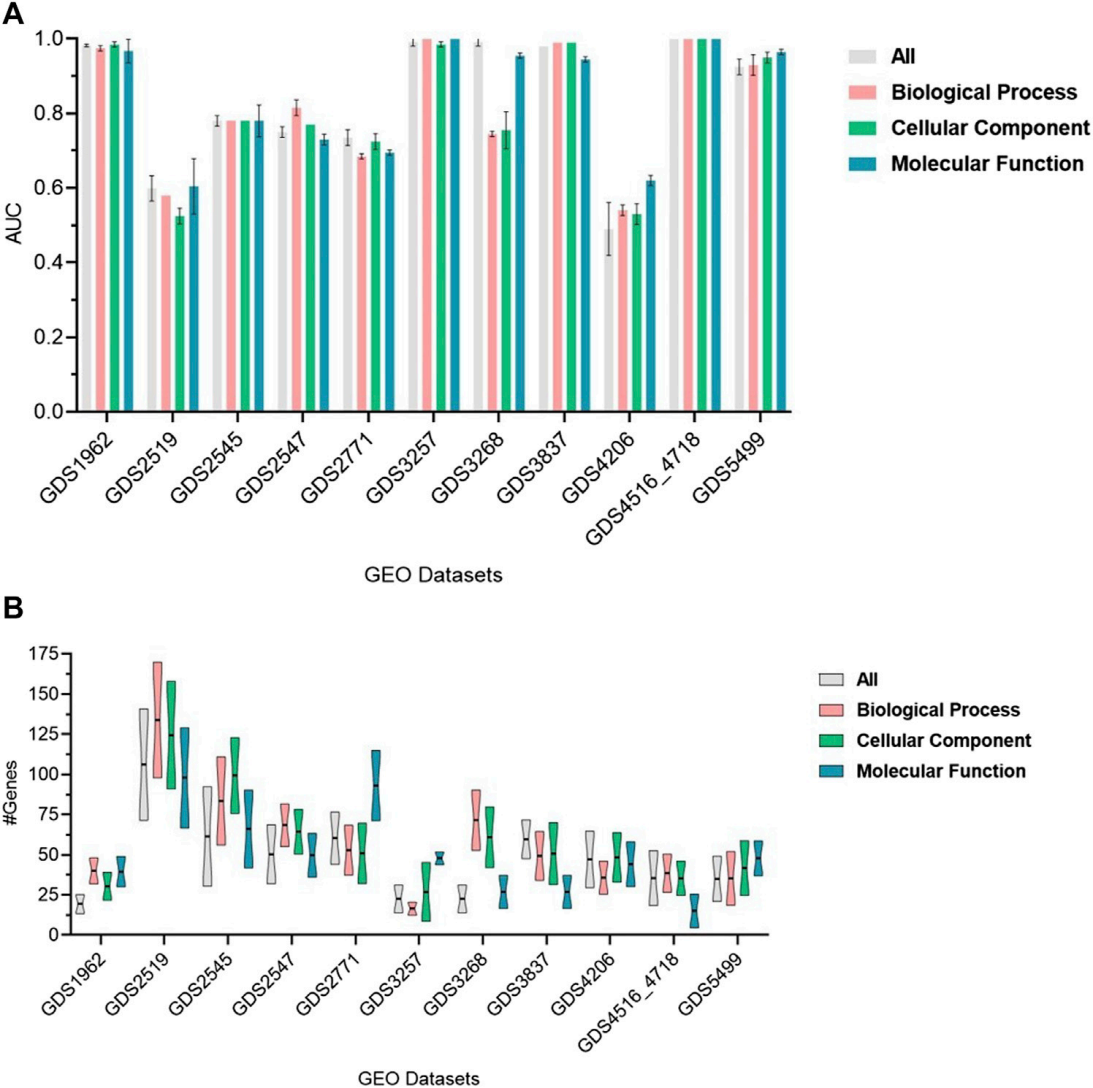
Performance results of GeNetOntology over the top-2-ranked groups with standard deviations for different datasets. **(A)** AUC and **(B)** average number of genes are plotted for GO BP, CC, and MF categories and all groups.

The performance of GeNetOntology over 11 datasets by using GO BP, CC, and MF categories and all GO terms are summarized in Figure 3. AUC is considered the performance metric, and all values in Figure 3 are the mean of 10-MCCV iterations, shown together with standard deviations. Figure 3A implies that except for the GDS3268, GDS2519, and GDS4206 datasets, the choice of GO category does not affect the performance in terms of AUC. Figure 3B implies that, except for the GDS2771, GDS3257, and GDS3268 datasets, the choice of the GO category does not affect the average number of genes in the top two selected GO terms by GeNetOntology.

### 3.2 Comparative performance evaluation of GeNetOntology with other GSM-based tools

**FIGURE 4:**
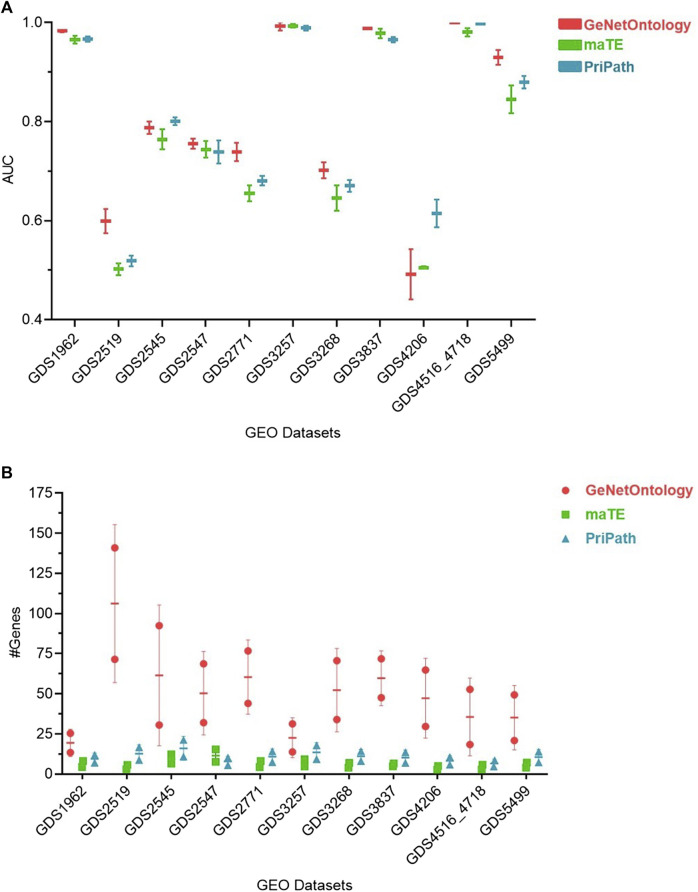
Comparative performance evaluations of GeNetOntology, maTE, and PriPath using 10-fold MCCV. **(A)** Mean AUC values; and **(B)** average number of genes are plotted for GeNetOntology, maTE, and PriPath results for 11 different datasets for top two scoring groups.

Pripath (Yousef et al., 2023) and maTE (Yousef et al., 2019) are two other G-S-M-based tools that incorporate biological domain knowledge. Although PriPath utilizes KEGG pathways as the biological knowledge, maTE uses miRNA information as pre-existing biological knowledge. maTE integrates information about miRNA target genes with gene expression data, and multiple high-scoring miRNAs are utilized while building the final classifier. PriPath tries to detect dysregulated pathways by using KEGG pathways as the grouping information and insert this information into an ML algorithm for selecting the most significant KEGG pathways in the gene expression dataset. We have compared GeNetOntology results with PriPath and maTE results (Figure 4). Although maTE, PriPath, and GeNetOntology generate distinct output tables, they all produce a table which demonstrates the tool’s performance. AUC performance metrics of GeNetOntology, PriPath, and maTE have been comparatively evaluated for 11 different datasets. We have considered the AUC values of the top two scoring sets for each tool by applying 10-fold MCCV. The mean AUC values of the three tools for the 11 datasets are represented in Figure 4A. One can deduce from Figure 4A that for GDS2519, GDS2771, and GDS5499 datasets, GeNetOntology performed higher than maTE and PriPath. For the remaining datasets, the AUC values are comparable. The mean number of genes utilized by the tools is also plotted in Figure 4B.

### 3.3 Biological validation of the GeNetOntology: analysis of the diagnostic model developed for the glioma dataset

In the previous section, we assessed the performance of GeNetOntology from a computational point of view using different computational performance evaluation metrics. In this section, we evaluate GeNetOntology findings from a biological point of view using the glioma dataset (GDS 1962).

#### 3.3.1 Correlation between top scored gene ontology terms for glioma dataset

**FIGURE 5:**
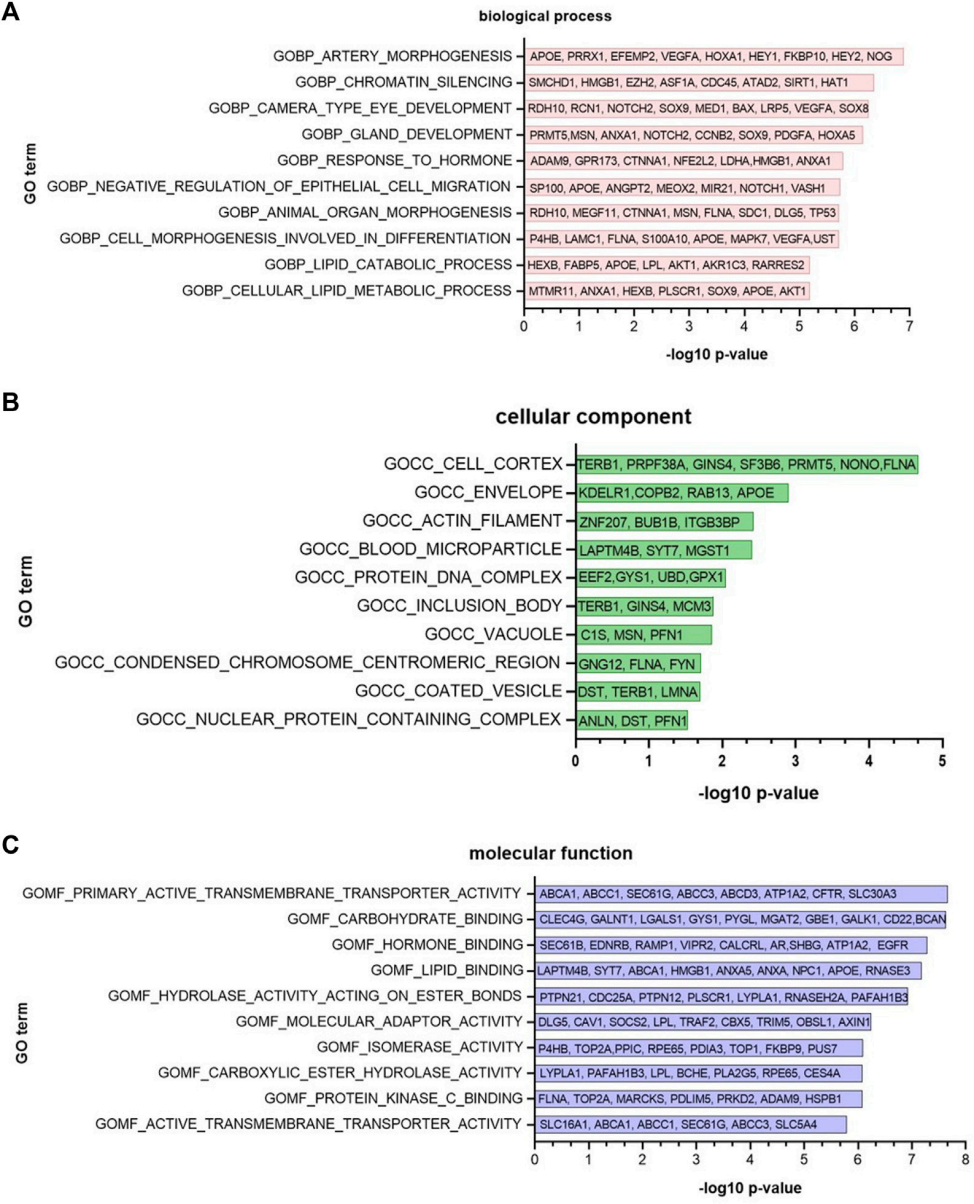
Top 10 important Gene Ontology terms in **(A)** BP, **(B)** CC, and **(C)** MF categories, identified by GeNetOntology for the GDS1962 glioma dataset. Although -log 10 *p*-values are represented on the *x*-axis, GO terms are represented on the *y*-axis, and some examples of the genes that belong to the associated GO terms are represented along the bars.

One of the outputs of the GeNetOntology tool is a list of ranked GO terms for the disease under study. The robust rank aggregation method of GeNetOntology calculates a *p*-value for each GO term, which shows the significance of the GO term in distinguishing the cases from controls for the disease under investigation. At the final step, the GO terms are ranked based on this *p*-value. In Figure 5, we plotted the top 10 important GO terms for the GDS1962 dataset associated with glioma. Although the robust rank aggregation *p* values are converted to -log 10 scale and shown in the *x*-axis, the GO terms are represented in the *y*-axis, and some examples of the genes annotated with the specified GO terms are represented on the bars. GO terms are ranked separately for BP, CC, and MF categories and shown in Figures 5A–C, respectively.

As depicted in Figure 5A, for the glioma dataset (GDS 1962), artery morphogenesis is the top ranked GO term in the BP category. Chromatin silencing, camera-type eye development, gland development, response to hormone, negative regulation of epithelial cell migration, animal organ morphogenesis, and cell morphogenesis involved in differentiation have similar *p* values; and lipid catabolic process and cellular lipid metabolic process have the lowest -log10 *p*-value among the top 10 ranked GO BP terms. As illustrated in Figure 5B, for the same dataset, the cell cortex has the highest score in the top 10 ranked GO CC category. Envelope, actin filament, blood microparticle, protein DNA complex, inclusion body, vacuole, condensed chromosome centromeric region, coated vesicle, and nuclear protein-containing complex are the other top ranked terms in the GDS1962 gene expression dataset. As shown in Figure 5C, primary active transmembrane transporter activity and carbohydrate binding GO terms have the highest importance for the GDS1962 gene expression dataset in the MF category. Hormone binding, lipid binding, hydrolase activity acting on ester bonds, molecular adapter activity, isomerase activity, carboxylic ester hydrolase activity, protein kinase c binding, and active transmembrane transporter activity are the other GO MF terms selected among the top 10 for the glioma dataset. These results guided us to apply further analysis to investigate the possible relationships between these GO terms.

**FIGURE 6:**
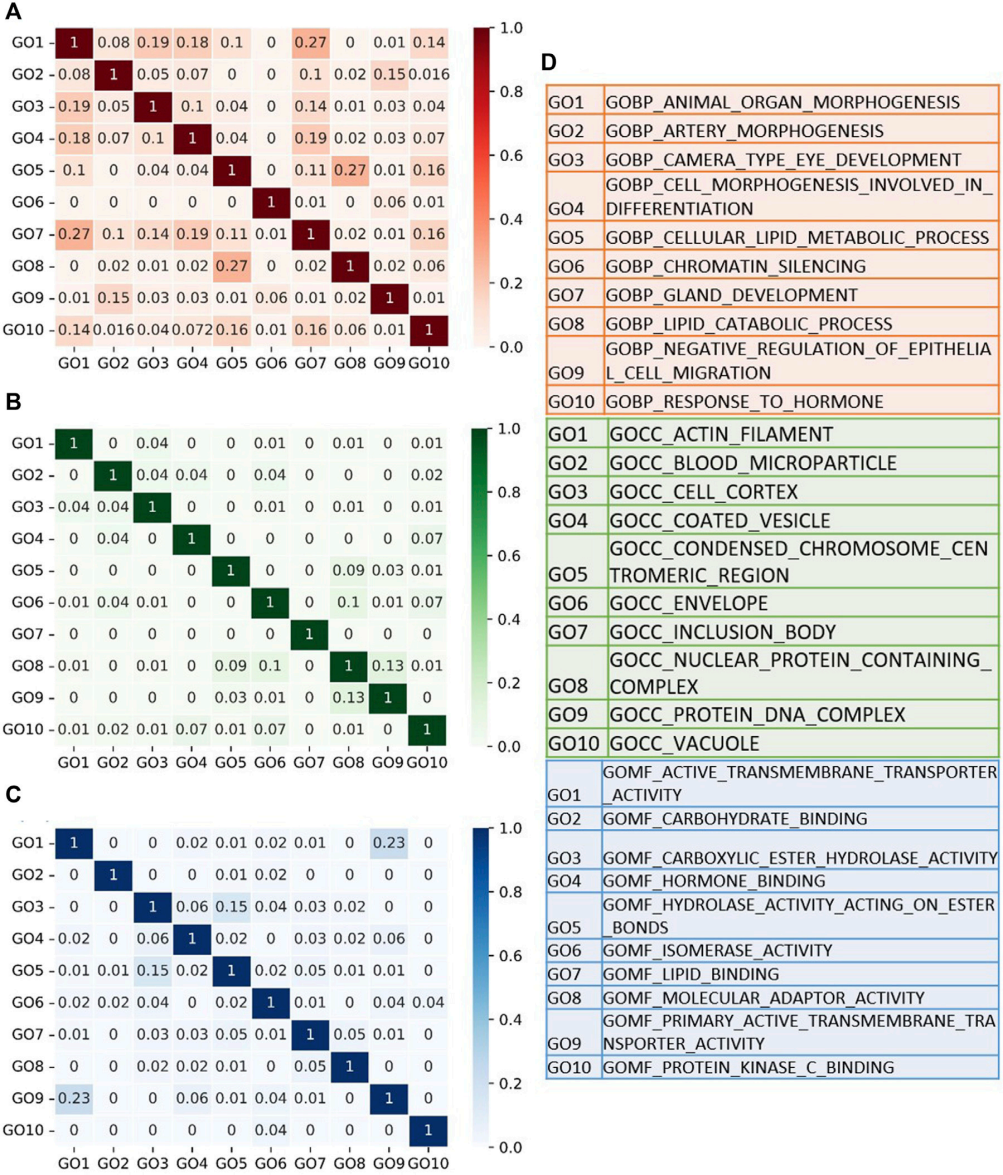
Correlations among the top 10 Gene Ontology terms identified by GeNetOntology for the GDS1962 glioma dataset. The pairwise correlations are calculated based on the number of shared genes within GO term pairs. Heatmaps for pairwise correlations of the GO terms are visualized in **(A)** BP; **(B)** CC; **(C)** and MF categories. **(D)** Full names of the GO terms.

It has been mentioned in literature that GO terms facilitate our understanding of the disease development and progression at the molecular level (Denny et al., 2018). Studying the associations between GO terms may further help us enlighten the relations of the GO terms with the disease mechanisms. In Figures 6A–C, for the GDS1962 glioma dataset, we present the pairwise correlations among the top 10 scoring gene ontology terms for BP, CC, and MF categories, respectively. In the BP category, organ morphogenesis and gland development terms have moderate correlations in terms of their shared genes) (as shown in a heatmap in Figure 6A). In the GO CC category, no significant correlation is observed between the top 10 identified GO terms (as depicted in Figure 6B). In the GO MF category, primary active transmembrane transporter activity and active transmembrane transporter activity have a moderate relationship (as displayed in Figure 6C). Figure 6 implies that the set of genes in each one of the top 10 scoring GO terms are nearly unique and there is minimum redundancy between the genes that are annotated with the selected GO terms. Hence, each selected GO term contributes to the classification. In other words, there are not so many overlapping genes in the top 10 selected GO terms. This finding is independent from the BP, CC, and MF categories, as shown with low or medium pairwise correlations in Figures 6A–C, respectively. Figure 6D shows the axis names of GO terms for BP, CC, and MF.

#### 3.3.2 PPI network analysis of the genes included in the top 10 scoring GO terms

**FIGURE 7:**
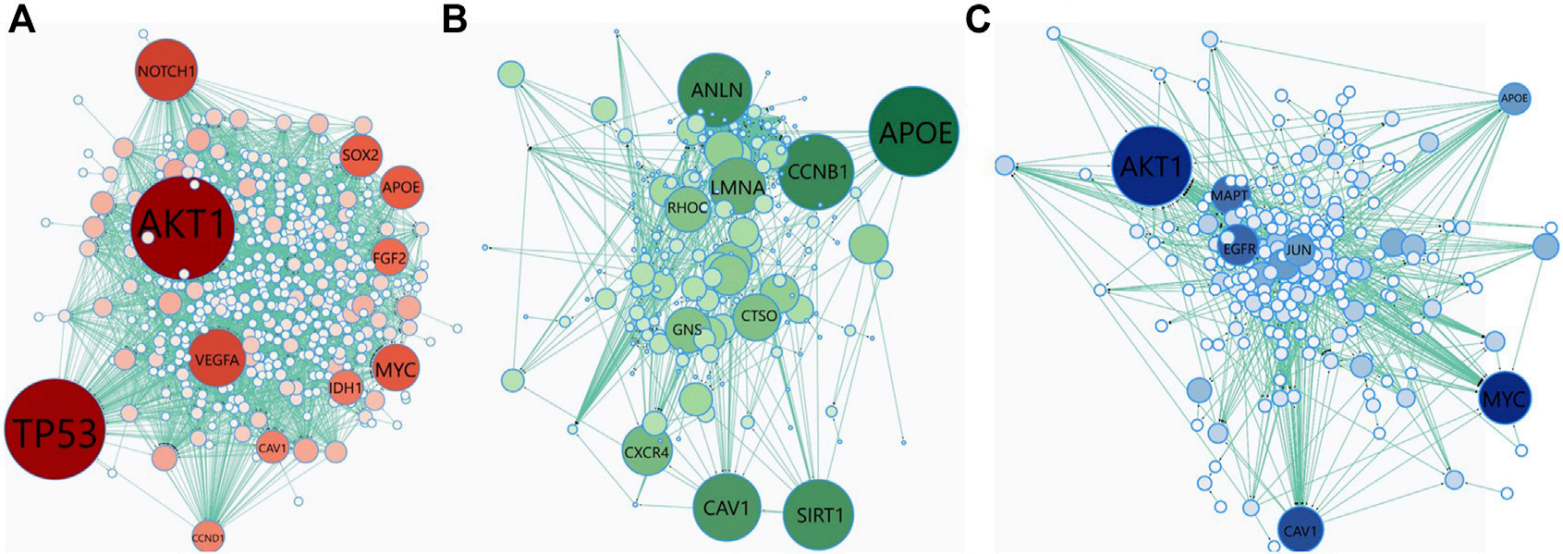
Protein–protein interaction (PPI) network of the genes included in the top-10 scoring GO terms detected by GeNetOntology for the GDS1962 dataset. Genes are collected from the top 10 GO terms in **(A)** BP; **(B)** CC; and **(C)** MF categories. Color and node size denote betweenness centrality. Bigger and darker colored nodes have higher betweenness centrality in the PPI network.

We have collected the genes that are annotated with the top 10 scoring GO terms identified by GeNetOntology for the GDS1962 glioma dataset. Then, we analyzed the topological properties of these proteins within the human PPI network. To this end, Figure 7 represents the PPI network of the genes annotated with the top 10 scoring GO terms detected by GeNetOntology for the GDS1962 dataset. We repeated this procedure separately for GO BP, CC, and MF categories and illustrated in Figures 7A–C, respectively. The PPI networks including 460 genes with 2,809 interactions for the BP category, 236 genes with 990 interactions for the MF category, and 284 genes with 777 interactions for e CC category are represented in Figures 7A–C, respectively. For each protein, we have computed the betweenness centrality which indicates the amount of control that this node exerts over the interactions of other nodes in the network (Yoon et al., 2006). Bigger and darker colored nodes in Figure 7 represent the proteins with higher betweenness centrality. One can easily observe from Figure 7A that TP53, AKT1, VEGFA, IDH1, MYC, APOE, NOTCH1, SOX2, FGF2, CAV1, and CCND1 have high betweenness centrality in the BP category for the glioma dataset. It implies that for glioma, these genes play key roles between other nodes (proteins) as connective proteins. In terms of the GO CC category, APOE, CCNB1, LMNA, RHOC, GNS, ANLN, CAV1, SIRT1, CTSO, and LMNA proteins are found to have high betweenness centrality in the analysis of GeNetOntology on the glioma dataset. One can imply from Figure 7B that these core proteins may play an important role for glioma. As visualized in Figure 7C, AKT1, MAPT, MYC, APOE, JUN, CAV1, and EGFR are hub proteins in the PPI network that are generated according to the top 10 scoring GO terms identified by GeNetOntology on the MF category for the glioma dataset.

#### 3.3.3 Frequency and word cloud analysis of the genes associated with top 10 gene ontology terms in the glioma dataset

**FIGURE 8:**
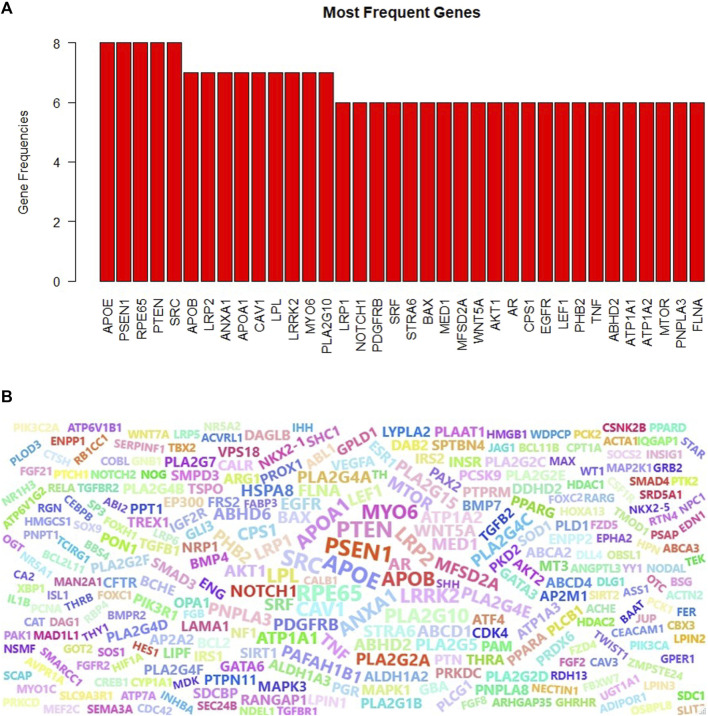
Most frequent genes for the GDS1962 dataset (associated with glioma cancer) in the top-10 scoring GO terms for the BP, CC, and MF categories **(A)**, word cloud of the top-10 scoring GO terms **(B)** are identified by GeNetOntology.

Proteins can play different roles in the organism, and hence they may be included in different GO terms. We have collected the genes that are annotated with the top 10 scoring GO terms identified by GeNetOntology for the GDS1962 glioma dataset. This time, instead of having three different gene sets for BP, CC, and MF categories, we merged these lists and obtained one gene set. In other words, if a gene is annotated with at least one of the top 10 scoring GO BP, CC, or MF terms for the glioma dataset, we include this gene into our final list. For the identified genes, we also keep track of the frequencies or how many times a gene is observed in any one of the top 10 scoring GO BP, CC, or MF terms for the glioma dataset (as shown partially in Figure 8A). One can observe from Figure 8A that APOE, PSEN1, RPE65, PTEN, and SRC have the highest frequencies. These five genes are annotated with eight GO terms among the top 10 scoring GO terms in any one of the BP, CC, and MF categories. Here, the top 10 scoring GO terms refer to the terms that are identified by GeNetOntology while analyzing the GDS1962 glioma dataset. We have also visualized the frequency of the genes from the top 30 scoring GO terms as a word cloud. To this end, Figure 8B presents the top 100 frequent genes from the top 30 scoring GO terms, where the top 10 scoring GO terms are identified by GeNetOntology for each one of the BP, CC, and MF categories for the glioma dataset.

## 4 Discussion

### 4.1 Computational performance evaluation of GeNetOntology

GeNetOntology is a novel and highly effective approach that predicts disease-causing genes by modeling and analyzing gene expression data with GO terms. In GeNetOntology, we have implemented an ML algorithm to select the most significant GO terms. Yet, when more terms are included, we did not obtain any statistically significant improvement in the performance metrics. Therefore, it is possible to construct a model with less number of genes, which facilitates the interpretation of the generated model. The GeNetOntology was tested on 11 different gene expression datasets, including cancer, hypertension, and other diseases. For the present analysis, we have tested GeNetOntology using i) all GO terms as all terms; ii) terms in BP; iii) in CC; and iv) in MF categories. Under these four settings, the performance results (in terms of AUC metric) using the top two scoring GO terms from different categories for different datasets are shown in Figure 3. In Figure 3, we present the mean of the AUC values for 10-fold cross-validations for all, BP, CC, and MF categories and the mean number of genes for each one of the 11 datasets.

As shown in Figure 3, GeNetOntology performs well on all datasets, except GDS2519 and GDS4206. When we analyzed different performance metrics of GeNetOntology over the top two ranked GO terms for different datasets, we noticed one more time that GeNetOntology performs well on all datasets, except GDS2519 and GDS4206, as shown in Table 6. In addition, when these datasets are analyzed with other tools such as maTE and PriPath, it has been observed that other tools also generated poor performance on these two datasets (as shown in Figure 4). As it can be seen in Supplementary Tables S2-S5, the AUC scores of all tested classifiers (Adaboost, DT, LogiBoost, RF, SMV_opt, Stack_Logitboost_Kmeans, and Stack_SVM_Kmeans) and all tested feature selection methods (XGB, IG, SKB, and FCBF) are also low for GDS2519 and GDS4206 datasets.

We have also tested for the effect of cumulatively adding top scoring GO terms (increasing the gene number) in terms of the performance of GeNetOntology. In Table 4, we present the performance metrics of GeNetOntology averaged over 10-fold MCCV iterations for the aggregated top-10 scoring GO terms for the GDS1962 dataset. For example, GeNetOntology has an AUC value of 0.97 when on average 31.9 genes are used, as shown in the last row of Table 4 (results of the top scoring GO term). On the other hand, the AUC value of GeNetOntology becomes 1 when 133.9 genes from the top-10 scoring GO terms are cumulatively used (as shown in the first row of Table 4). Instead of checking the gene expression values of 134 genes, one can prefer to check the expression values for only 32 genes to predict whether the sample has glioma while sacrificing 3% of AUC. To put it another way, the model that is generated via only using the gene expression values of 32 genes can successfully predict glioma patients with an AUC score of 0.97, which is quite a satisfying result.

Additionally, GeNetOntology is comparatively evaluated with other G-S-M-based tools. For 11 different gene expression datasets, the mean AUC values of 10-fold cross-validations for GeNetOntology, maTE, and PriPath were compared using the genes from the top two scoring feature sets. One can obtain from Figure 4A that for GDS2519, GDS2771, and GDS5499 datasets, GeNetOntology performed better than maTE and PriPath. For the remaining datasets, the AUC values were comparable. On the other hand, the outcome and contribution of each tool is different because each of these tools aims to identify important feature sets via using a different biological knowledge. For example, while defining important feature sets, PriPath makes use of KEGG pathway information, and the final output of PriPath is the top scoring pathways and their associated genes. In maTE, miRNA–target gene information is utilized, and the final outcome is top scoring miRNAs and their associated genes. However, GeNetOntology exploits GO as biological knowledge; and as an output, it identifies top scoring GO terms and their associated genes that can best distinguish patients from healthy samples for the specific disease under study.

### 4.2 Biological evaluation of GeNetOntology findings

#### 4.2.1 Top scoring gene ontology terms for the glioma dataset

As the performance metrics shown in Table 6, Figure 3A, Figure 4A implies, GeNetOntology is capable of discovering significant gene ontology terms that can perform as a marker for classification. In the previous section, GeNetOntology is comparatively evaluated with other G-S-M-based tools. Here, we evaluated whether the top scoring GO terms identified by GeNetOntology are supported by previous experimental research.

For the glioma dataset, the top 10 significant GO BP terms include morphogenesis, development, and differentiation (Figures 6A, D). In the literature, it has been shown that morphogenesis is highly correlated with cancer invasion to other tissues. Lipids are a complex group of biomolecules, and they form the main structure of biological membranes, an energy source, and also act as signaling molecules (Snaebjornsson et al., 2020). Lipid metabolism has an impact on cancer formation and promotion since lipid metabolism, carcinogenesis, and cancer metastasis are related with abnormal levels of lipids (Jiang et al., 2020). Chromatin is a critical and dynamic major regulator of transcription. Studies showed that deregulation of chromatin guides gene activation alteration and/or improper gene silencing and is able to promote oncogenesis by altering chromatin structures (Nair and Kumar, 2012). Numerous studies have demonstrated that epigenetic gene silencing is a critical mechanism for the loss of gene function in many cancers (Baylin and Ohm, 2006). Several GO BP terms such as tissue formation, embryological development, inflammation, immune defense, and cancer progression, are highly related with cell migration (Pijuan et al., 2019). Unusual regulation of epithelial cell migration has a significant role in pathological processes such as cancer metastasis and tissue fibrosis development (Magliozzi et al., 2013). Cancer cell invasion mechanisms and metastasis based on cellular motility is a complicated process (Lorente et al., 2014). An essential characteristic of tumor cell invasion and metastasis is the ability of improved motility and migration capability of the tumor cells. It is important to understand the specific mechanisms of cellular motility in cancer development because a loss or deficiency of the mechanisms that regulate cytoskeletal remodeling might result in tumor development and metastasis (Lorente et al., 2014). Therefore, actin filament formation and cell cortex and envelope formation could potentially have a role in cancer formation. These terms are identified by GeNetOntology among top 10 significant GO CC terms, as shown in Figures 6B, D. Blood microparticles originate from the endothelial lining of blood vessels and cellular components of blood. Almost all cells, when they are exposed to several stress conditions such as apoptosis and cellular activation, shed parts of their plasma membranes, and these are called cellular microparticles (MPs) (Shet, 2008). DNA packaging and gene expression regulation are performed by chromatin, which is a protein–DNA complex (de Brot et al., 2018). For the glioma dataset, the top-10 scoring GO MF terms of GeNetOntology include carbohydrate-, lipid-, and hormone-binding molecular functions which regulate several intracellular and extracellular signaling pathways (Figures 6C, D).

#### 4.2.2 Top scoring genes for the glioma dataset

We have collected the genes that are annotated with the top 10 scoring GO terms identified by GeNetOntology for the GDS1962 glioma dataset. As shown in Figures 7A–C, several well-known cancer driver genes are present in the PPI network generated by using the genes from the top scoring GO BP, CC, and MF terms, respectively. TP53 is a well-known oncogene, and it is highly correlated with many cancer subtypes. AKT1 plays a vital role in many signaling pathways and in growth factor-induced neuronal survival during nervous system development (Dudek et al., 1997). VEGFA is a critical modulator of angiogenesis, and it has been shown in the literature that VEGFA expression is high in cancer tissue, and this is correlated with its aggressive characteristics (Sa-nguanraksa and O-charoenrat, 2012). Mutation in the IDH1 gene has been found in many genetic conditions and cancer types, such as acute myeloid leukemia, glioblastoma, and myelodysplastic syndromes (Dang et al., 2016). MYC is a proto-oncogene, and it is overexpressed in several tumors. It can escape from several tumor-suppressing checkpoint mechanisms such as apoptosis senescence, proliferative arrest, and induces tumorigenesis (Li et al., 2014). In several tumors, APOE overexpression is related to poor prognosis and aggressive biological behaviors (Zhao et al., 2018). NOTCH1 is associated with numerous signaling pathways in tumorigenesis, and it is involved in many types of cancer, including brain tumors, leukemia, breast cancer, and several other cancer types (Gharaibeh et al., 2020). SOX2 is dysregulated during gene amplification and promotes metastasis, drug resistance, and survival. Therefore, its overexpression is associated with a poor survival rate in cancer patients (Zhang et al., 2020). FGF2 gene expression is correlated with several cancer types, including colorectal cancer (Caiado et al., 2020). With their roles in top 10 scoring GO BP terms, these genes are emphasized in our analysis on the glioma dataset, and these genes are shown with bigger and darker nodes in Figure 7A based on their high betweenness centrality.

APOE plays an important role in GO CC as well (Figure 7B). CAV1 functions both as a tumor suppressor and metastasis promoter membrane-associated scaffolding protein, and it has shown that CAV1 is downregulated in human tumors (Díaz et al., 2020). CCND1, known as a proto-oncogene, switches to proximal APA sites in cancer cells and acts as the G1-S phase of the cell cycle regulator (Wang et al., 2018). It has been shown that in lung cancer, breast cancer, cervical cancer, and melanoma and esophageal squamous cell carcinoma, CCNB1 expression is relatively high (Li et al., 2019). LMNA functions as an oncogene in many cancer cell types, especially in hepatocellular carcinoma (Liu et al., 2020). Understanding the role of RhoC-regulated migration processes is crucial to deal with cancer metastasis mechanisms (Lou et al., 2021). In many types of site-specific cancerous tumors, such as bone marrow, brain, breast, colorectal, pancreas, and lung cancer, ANLN is highly expressed (Hall et al., 2005; Olakowski et al., 2009; Uhlén et al., 2015; Tuan and Lee, 2020). Several studies have presented that SIRT1 can function as a tumor promoter or tumor suppressor depending on its targets in specific cancer and signaling pathways (Lin and Fang, 2013). CTSO is a biomarker that can predict which women will emanate the highest benefit from a selective estrogen receptor modulator (SERM) therapy (Brentnall et al., 2016). With their roles in top 10 scoring GO CC terms, these genes are emphasized in our analysis on the glioma dataset, and these genes are shown with bigger and darker nodes in Figure 7B based on their high betweenness centrality.

AKT1, MAPT, MYC, APOE, JUN, CAV1, and EGFR are identified as hub proteins in the PPI network in Figure 7C, which is generated using the proteins associated with the top 10 scoring GO MF terms. The associations of most of these genes with glioma are also reported in literature as follows. Tauopathies, known as neurodegenerative disorders, are characterized by abnormal tau protein deposition in the brain, and MAPT expression is a biomarker for tauopathies and an increased survival rate and low-grade glioma (Zaman et al., 2019). JUN is a proto-oncogene transcription factor and regulates transcription-caused cancer formation (Expression of JUN in cancer - Summary - The Human Protein Atlas, 2021). EGFR is a driver of tumorigenesis and is identified as a biomarker of resistance in tumors, especially in glioblastoma, breast, and lung cancer (Sigismund et al., 2018). In the literature, it has been shown that the APOE gene is related with tumorigenesis and progression, such as cell proliferation, angiogenesis, and metastasis (Zhao et al., 2018; Adaku et al., 2022).

#### 4.2.3 The most frequent genes and word cloud of the genes associated with top 10 GO terms in the glioma dataset

Genes might have several functions in the living organism and act with multiple roles. Therefore, a gene can be annotated with different GO terms. When a gene is mutated, gain or lose function, several biological processes and molecular functions would be affected. To this end, we analyzed the frequency of the genes that are annotated with the top 10 scoring ontology terms. Figure 8A showed that APOE, PSEN1, PTEN, RP65, and SRC genes play a role in eight different top scoring GO terms for the glioma dataset. Although the importance of the APOE gene for glioma is discussed in the aforementioned section, the other frequently observed genes are potentially associated with glioma as follows. PSEN1 gene missense mutation is a well-known cause of a neurological disorder, Alzheimer’s disease (Randa, 2019). The RPE65 gene mutation causes several inherited retinal diseases because it provides instructions to make essential proteins for normal vision (Sodi et al., 2021; *RPE65 gene: MedlinePlus Genetics*). PTEN can function as a tumor suppressor in a PI3K signaling pathway, and when the tumor suppressor function of the PTEN enzyme is disrupted by mutations, it causes cells to grow and uncontrolled division and contributes to a cancerous tumor formation (Milella et al., 2015). Several human cancers, such as colorectal, lung, breast, and prostate cancer, have been strongly related to SRC, which promotes maintenance, progression, development, and metastasis of cancers (Wheeler et al., 2009). We extended the most frequently observed gene list and compiled the list of the top 100 genes which are annotated with the top 10 GO terms identified by GeNetOntology for the glioma dataset. The word cloud visualized in Figure 8B shows that this list included several glioma-associated genes, where several of these genes are discussed in the previous section. Taken together with our previous results, GeNetOntology findings imply that the identified genes have a significant impact on disease development and progression for glioma.

## 5 Conclusion

The current advancements in next-generation sequencing and other high-throughput technologies make it possible to acquire gene expression profiles from tissue samples at quite low expenses. Various gene expression datasets were publicly available right after these technologies were developed, and extracting knowledge from these datasets became a major challenge. In this study, we have introduced a computational tool that uses biological knowledge from GO, which is implemented into the ML algorithm performing gene selection. Our methodology is different from the standard approaches where the analysis is carried out by considering individual genes; however, GeNetOntology has focused on the investigation of the ontology terms to rank and discover the most influential feature sets. Performance evaluations over 11 different datasets showed that the GeNetOntology tool is consistent and robust. We have compared the performance of GeNetOntology with that of PriPath and maTE, which are similar in their merits. The results show that, in most cases, GeNetOntology outperforms maTE and PriPath, depending on the gene expression dataset. We believe that GeNetOntology will assist scientists, medical geneticists, and physicians in studying and analyzing their gene expression datasets and in better understanding disease-related genes and the main mechanisms behind disease development and progression. GeNetOntology could help researchers define dysregulated genes and gene ontology terms in BP, CC, and MF categories, which can be potentially applied to medical diagnostics. As a future work, we intend to make improvements in our proposed approach in a way that allows us to perform patient stratification based on gene expression, and it allows us to determine druggable targets toward precision medicine.

## Data Availability

The GeNetOntology Knime workfow is freely available: https://github.com/malikyousef/GeNetOntology.git. All the datasets used in this study are publicly available at the gene omnibus at NCBI and can be retrieved using the cited accession numbers. GEO accession numbers of datasets (GDS1962, GDS2519, GDS2545, GDS2547, GDS2771, GDS3257, GDS3268, GDS3837, GDS4206, GDS4516_4718, GDS5499) are shown in the second column of Table 1.
