# Supplementary figures and images for: GeNetOntology: identifying affected gene ontology terms via grouping, scoring, and modeling of gene expression data utilizing biological knowledge-based machine learning
Source: Front Genet. 2023 Aug 21;14:1139082. doi: 10.3389/fgene.2023.1139082 (PMC10476493; doi:10.3389/fgene.2023.1139082)

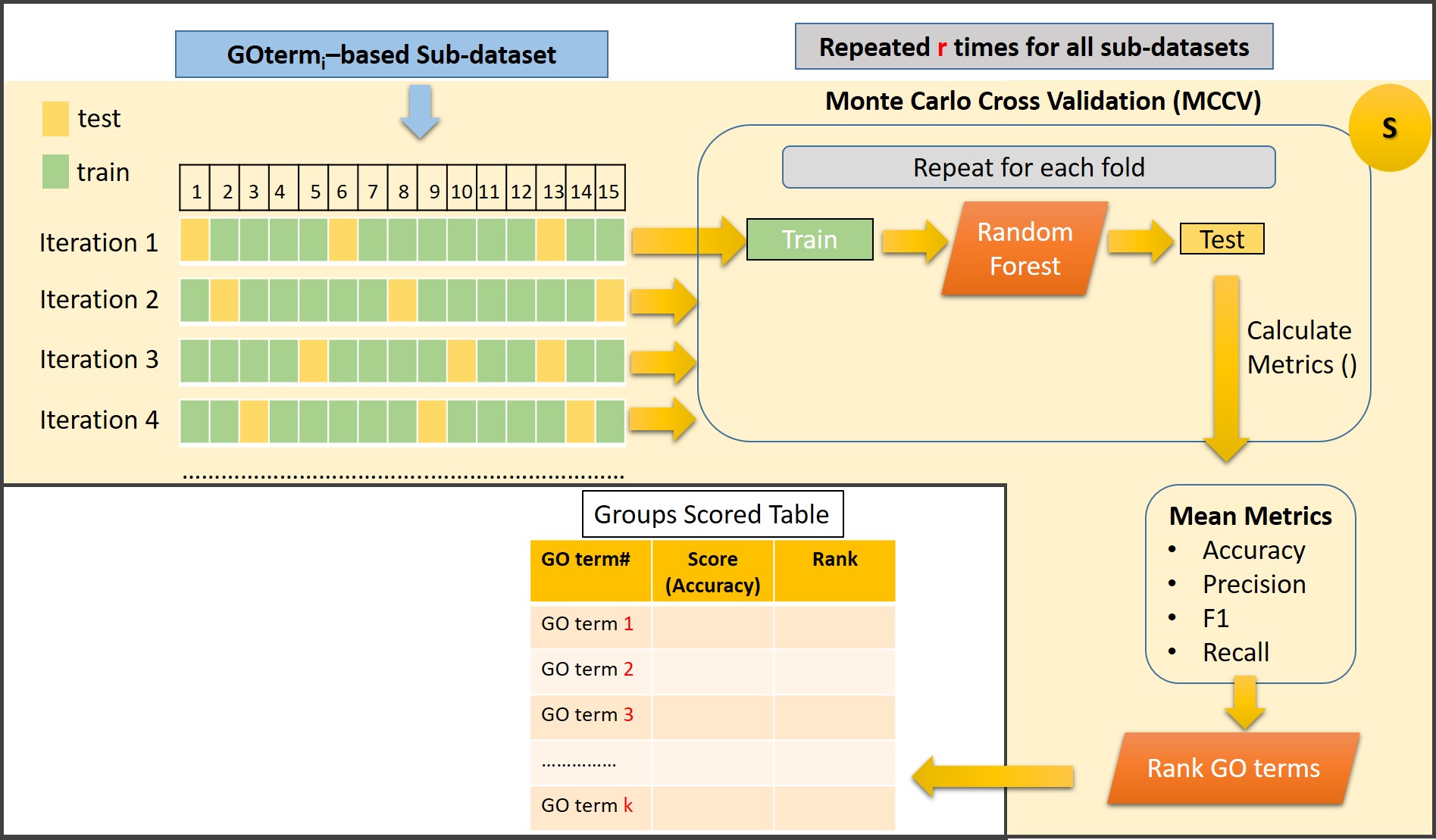

Supplement: Supplementary file 1 [file Image3.JPEG]

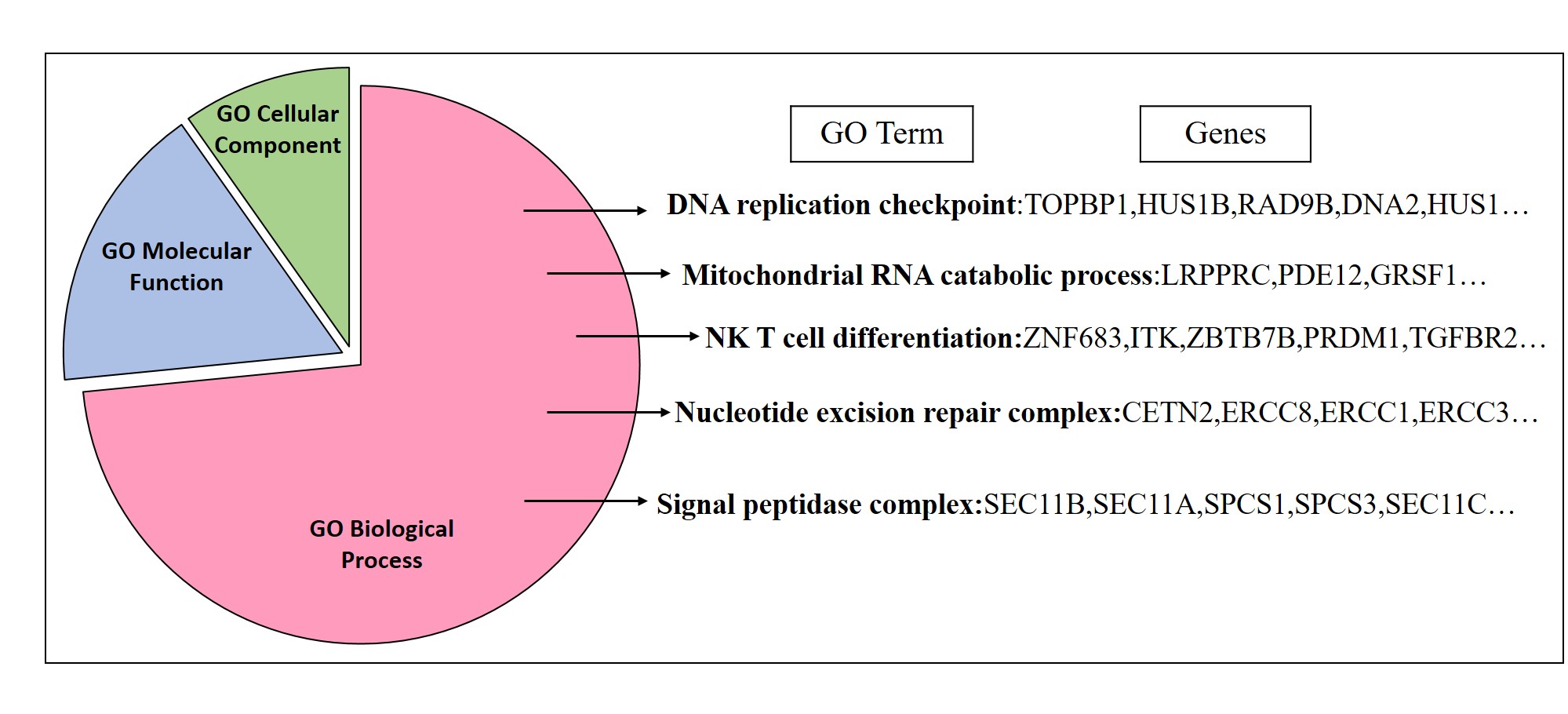

Supplement: Supplementary file 3 [file Image1.JPEG]

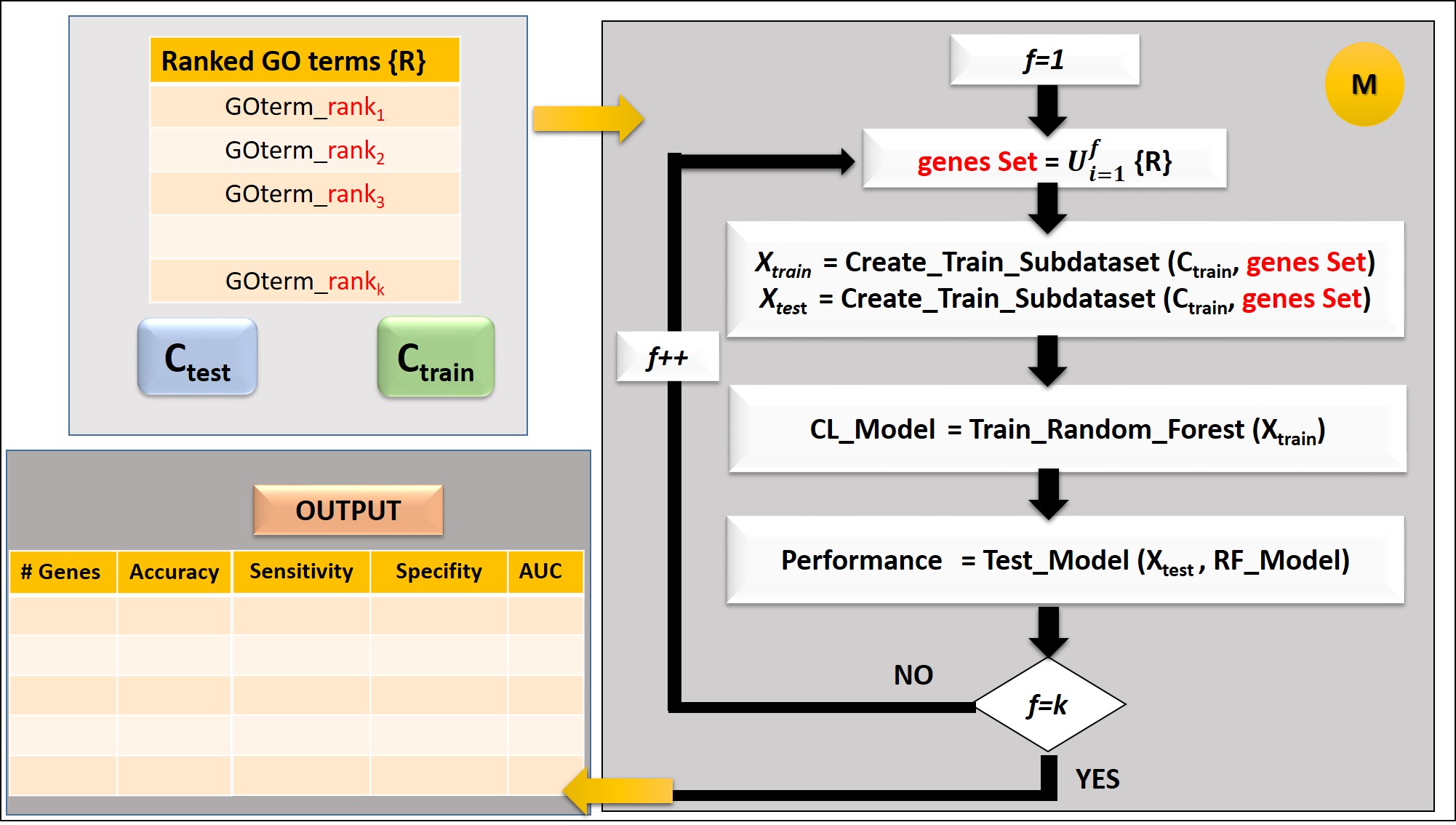

Supplement: Supplementary file 4 [file Image4.JPEG]

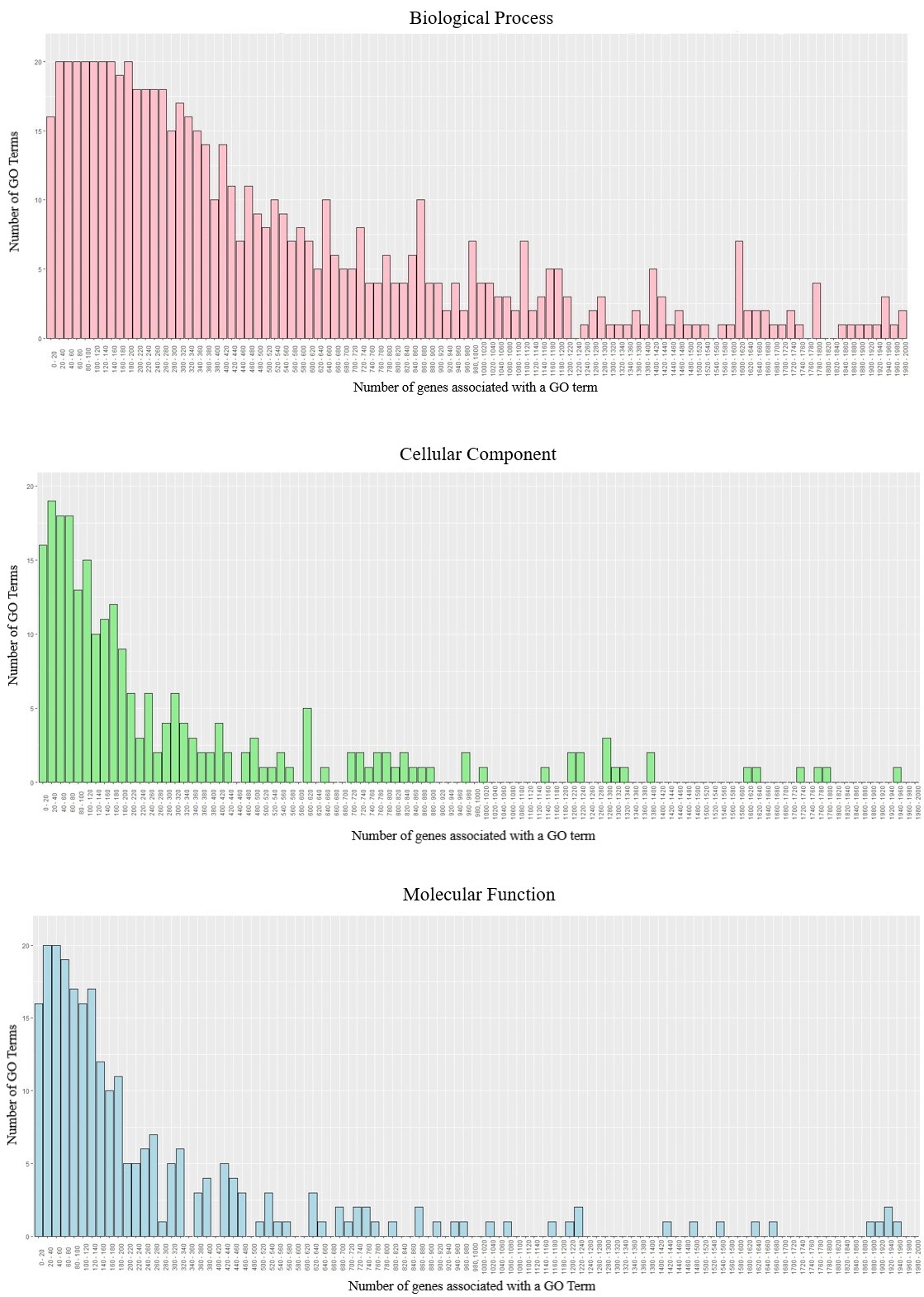

Supplement: Supplementary file 5 [file Image2.JPEG]

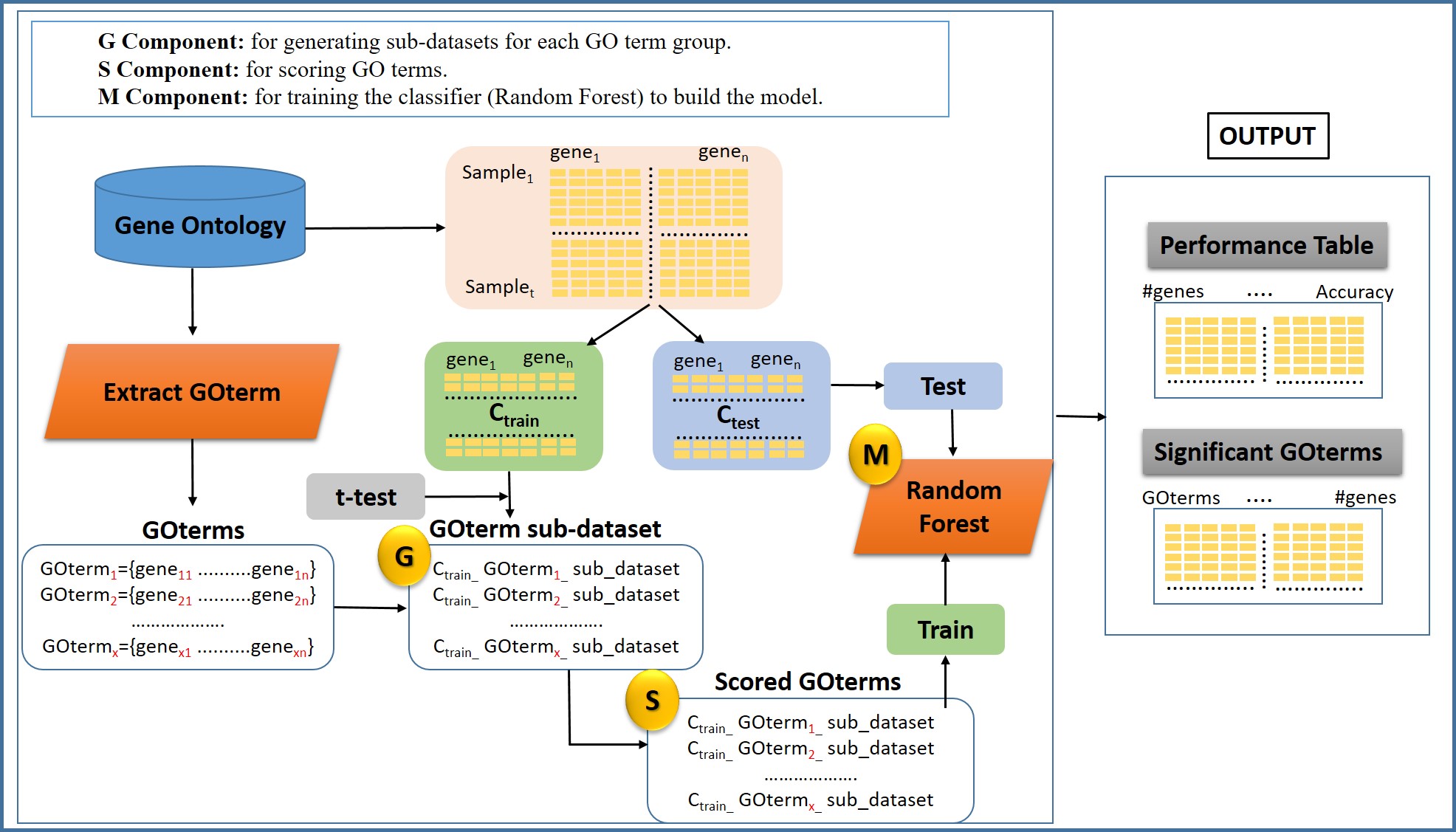

Supplement: Supplementary file 6 [file Image5.JPEG]

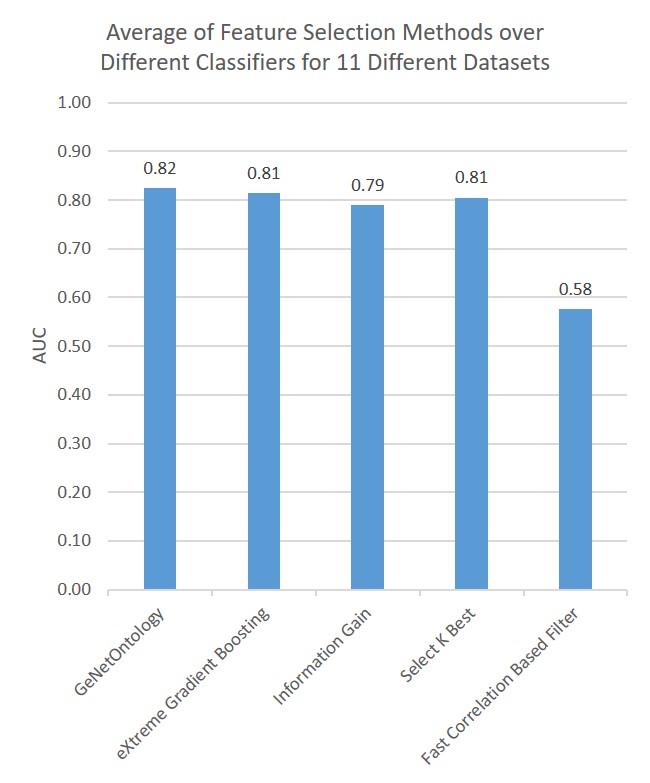

Supplement: Supplementary file 7 [file Image6.JPEG]
