# Supplementary material for: GeNetOntology: identifying affected gene ontology terms via grouping, scoring, and modeling of gene expression data utilizing biological knowledge-based machine learning
Source: Front Genet. 2023 Aug 21;14:1139082. doi: 10.3389/fgene.2023.1139082 (PMC10476493; doi:10.3389/fgene.2023.1139082)
Supplement: Supplementary file 2 [file Table1.DOCX]

**Supplementary Table 1**. GeNetOntology pseudo-code

**GeNetOntology pseudo-code**

**Input**

X: Two-class gene expression data, the features are expression values of the genes.

M {$\boldsymbol{m}_{\boldsymbol{1}},\boldsymbol{m}_{\boldsymbol{2}},\ldots,\boldsymbol{m}_{\boldsymbol{p}}\}$: list of *p* ontology groups extracted by G-component

**Algorithm**

M^*^={} empty list

Perform N-fold cross validation (here N = 10):

Randomly split data by samples into train (X_t_) and test (X_v_) parts,

performs steps 1- 6:

1. X_tt_ = filter genes (features) from training data by t-test (here p-value ≤ 0.05 and maximum number of filtered genes≤ 2000)
2. X_vf_ = remove all genes from X_v_ that are not in X_tf_
3. *grps_p_* = Scoring Component $(\boldsymbol{X}_{\boldsymbol{tf}},\boldsymbol{M},\boldsymbol{f}=\boldsymbol{0}.\boldsymbol{8},\boldsymbol{r}=\boldsymbol{5})$
4. M^*^= Sort( *grps_p_*) according to performance; best first
5. M^*^ = {m^*^_1_, m^*^_2_,..,m^*^_j_} , Select best *j* groups (here *j=2*)
6. Filter X_tf_ and X_vf_ by *g*(M^*^ ),now X_tf_ and X_vf_ represented by genes that are from M^*^.

Train classifier using X_tf_ and X_vf_

Test classifier using X_vf_

**Output**

Report performance (e.g.: average accuracy)

**Scoring Component** $(\boldsymbol{X}_{\boldsymbol{s}},M,\boldsymbol{f},\boldsymbol{r})$

**X_s_**: any subset of the input gene expression data X, the features are gene expression values

M {$\boldsymbol{m}_{\boldsymbol{1}},\boldsymbol{m}_{\boldsymbol{2}},\ldots,\boldsymbol{m}_{\boldsymbol{p}}\}$ is a list of groups

***f*** *is a scalar ($0\leq f\leq1$ )*: split into train and test data

**r**: repeated times (iteration)

res={} for aggregation the scores for each *m_i_*

/*Compute Score for each *m_i_, Score(m_i_) */*

**For each *m_i_* in M**

Extract sub dataset: Represent X_s_ using just genes from *m_i_* Ontology group.

*sm_i_*=0;

**Perform *r* time (here r=5) steps 1-5:**

1. Perform random sampling to split X_s_ into train X_t_ and test X_v_ according to *f* (here 80:20)
2. Remove all genes (features) from X_t_ and X_v_ which are not in the group *m_i_*
3. Train classifier on X_t_ (could Random Forest, SVM)
4. *t* = Test classifier on X_v_ –calculate performance
5. *sm_i_ = sm_i_ + t;*

*Score(m_i_)*= *sm_i_* /*r* ; Aggregate performance

*res*= $\bigcup_{i=1}^{p} Score(m_{i})$

**Output**

*Return res ( res = {Score(m_1_),Score(m_2_),…,Score(m_p_)} )*

**Supplementary Table 2**. Performance metrics obtained using eXtreme Gradient Boosting (XGB) feature selection method and different classifiers applied on 11  different gene expression datasets.

| Datasets | GDS1962 | GDS2519 | GDS2545 | GDS2547 | GDS2771 | GDS3257 | GDS3268 | GDS3837 | GDS4206 | GDS4516_4718 | GDS5499 |
| --- | --- | --- | --- | --- | --- | --- | --- | --- | --- | --- | --- |
| # of Genes\  Classifier | 50 | 170 | 110 | 80 | 70 | 20 | 90 | 65 | 50 | 50 | 55 |
| Adaboost | 0.98 | 0.51 | 0.81 | 0.86 | 0.73 | 0.95 | 0.88 | 0.98 | 0.61 | 1 | 0.97 |
| DT | 0.84 | 0.44 | 0.62 | 0.62 | 0.63 | 0.94 | 0.61 | 0.89 | 0.53 | 1 | 0.77 |
| LogitBoost | 0.98 | 0.46 | 0.8 | 0.88 | 0.77 | 0.95 | 0.86 | 0.97 | 0.58 | 1 | 0.96 |
| RF | 0.99 | 0.51 | 0.81 | 0.87 | 0.75 | 0.99 | 0.85 | 0.97 | 0.58 | 1 | 0.97 |
| SVM_opt | 0.96 | 0.55 | 0.8 | 0.82 | 0.7 | 1 | 0.85 | 0.98 | 0.59 | 1 | 0.98 |
| Stack_  Logitboost_  Kmenas | 0.93 | 0.47 | 0.73 | 0.8 | 0.71 | 0.95 | 0.85 | 0.95 | 0.55 | 1 | 0.95 |
| Stack_  SVM_  Kmeans | 0.92 | 0.49 | 0.8 | 0.82 | 0.74 | 0.98 | 0.86 | 0.91 | 0.5 | 1 | 0.97 |
| average | 0.94 | 0.49 | 0.77 | 0.81 | 0.72 | 0.97 | 0.82 | 0.95 | 0.56 | 1.00 | 0.94 |

**Supplementary Table 3**: Performance metrics obtained using Information Gain (IG) feature selection method and different classifiers applied on 11 different gene expression datasets.

|  | GDS1962 | GDS2519 | GDS2545 | GDS2547 | GDS2771 | GDS3257 | GDS3268 | GDS3837 | GDS4206 | GDS4516_4718 | GDS5499 |
| --- | --- | --- | --- | --- | --- | --- | --- | --- | --- | --- | --- |
| # of Genes\  Classifier | 50 | 170 | 110 | 80 | 70 | 20 | 90 | 65 | 50 | 50 | 55 |
| Adaboost | 0.94 | 0.66 | 0.78 | 0.74 | 0.65 | 0.98 | 0.73 | 0.98 | 0.45 | 1 | 0.86 |
| DT | 0.83 | 0.5 | 0.66 | 0.63 | 0.6 | 0.95 | 0.55 | 0.93 | 0.5 | 1 | 0.82 |
| LogitBoost | 0.99 | 0.59 | 0.8 | 0.77 | 0.68 | 0.98 | 0.76 | 0.99 | 0.6 | 1 | 0.93 |
| RF | 0.99 | 0.56 | 0.79 | 0.74 | 0.69 | 1 | 0.75 | 0.98 | 0.56 | 1 | 0.92 |
| SVM_opt | 0.98 | 0.41 | 0.67 | 0.75 | 0.72 | 1 | 0.66 | 0.99 | 0.58 | 1 | 0.83 |
| Stack_  Logitboost_  Kmenas | 0.99 | 0.51 | 0.72 | 0.74 | 0.62 | 0.99 | 0.62 | 0.97 | 0.52 | 1 | 0.89 |
| Stack_  SVM_  Kmeans | 0.99 | 0.55 | 0.77 | 0.73 | 0.69 | 1 | 0.76 | 0.98 | 0.47 | 1 | 0.87 |
| average | 0.96 | 0.54 | 0.74 | 0.73 | 0.66 | 0.99 | 0.69 | 0.97 | 0.53 | 1.00 | 0.87 |

**Supplementary Table 4**: Performance metrics obtained using Select K Best (SKB) feature selection method and different classifiers applied on 11 different gene expression datasets.

|  | GDS1962 | GDS2519 | GDS2545 | GDS2547 | GDS2771 | GDS3257 | GDS3268 | GDS3837 | GDS4206 | GDS4516_4718 | GDS5499 |
| --- | --- | --- | --- | --- | --- | --- | --- | --- | --- | --- | --- |
| # of Genes\  Classifier | 50 | 170 | 110 | 80 | 70 | 20 | 90 | 65 | 50 | 50 | 55 |
| Adaboost | 0.98 | 0.58 | 0.76 | 0.87 | 0.71 | 0.96 | 0.78 | 1 | 0.54 | 1 | 0.92 |
| DT | 0.79 | 0.46 | 0.67 | 0.67 | 0.64 | 0.95 | 0.62 | 0.96 | 0.57 | 1 | 0.81 |
| LogitBoost | 0.93 | 0.49 | 0.79 | 0.85 | 0.71 | 0.97 | 0.77 | 1 | 0.55 | 1 | 0.96 |
| RF | 0.95 | 0.59 | 0.86 | 0.86 | 0.75 | 0.99 | 0.78 | 1 | 0.68 | 1 | 0.95 |
| SVM_opt | 0.93 | 0.55 | 0.75 | 0.83 | 0.63 | 0.99 | 0.8 | 1 | 0.66 | 1 | 0.91 |
| Stack_  Logitboost_  Kmenas | 0.87 | 0.47 | 0.69 | 0.81 | 0.64 | 0.97 | 0.73 | 0.99 | 0.52 | 1 | 0.94 |
| Stack_  SVM_  Kmeans | 0.85 | 0.47 | 0.48 | 0.82 | 0.73 | 1 | 0.82 | 1 | 0.56 | 1 | 0.93 |
| average | 0.90 | 0.52 | 0.71 | 0.82 | 0.69 | 0.98 | 0.76 | 0.99 | 0.58 | 1.00 | 0.92 |

**Supplementary Table 5**: Performance metrics obtained using Fast Correlation Based Filter (FCBF) feature selection method and different classifiers applied on 11 different gene expression datasets.

|  | GDS1962 | GDS2519 | GDS2545 | GDS2547 | GDS2771 | GDS3257 | GDS3268 | GDS3837 | GDS4206 | GDS4516_4718 | GDS5499 |
| --- | --- | --- | --- | --- | --- | --- | --- | --- | --- | --- | --- |
| # of Genes\  Classifier | 50 | 170 | 110 | 80 | 70 | 20 | 90 | 65 | 50 | 50 | 55 |
| Adaboost | 1 | 0.5 | 0.5 | 0.5 | 0.49 | 0.57 | 0.54 | 0.34 | 0.44 | 0.68 | 0.5 |
| DT | 0.75 | 0.54 | 0.56 | 0.46 | 0.49 | 0.46 | 0.53 | 0.5 | 0.52 | 0.64 | 0.49 |
| LogitBoost | 1 | 0.56 | 0.55 | 0.55 | 0.5 | 0.55 | 0.51 | 0.54 | 0.53 | 0.72 | 0.42 |
| RF | 1 | 0.55 | 0.51 | 0.57 | 0.51 | 0.53 | 0.51 | 0.63 | 0.46 | 0.77 | 0.52 |
| SVM_opt | 1 | 0.55 | 0.55 | 0.51 | 0.46 | 0.53 | 0.43 | 0.58 | 0.5 | 0.93 | 0.43 |
| Stack_  Logitboost_Kmenas | 0.94 | 0.44 | 0.57 | 0.53 | 0.48 | 0.51 | 0.5 | 0.59 | 0.47 | 0.94 | 0.5 |
| Stack_  SVM_  Kmeans | 0.75 | 0.46 | 0.62 | 0.52 | 0.54 | 0.59 | 0.52 | 0.49 | 0.48 | 0.96 | 0.52 |
| average | 0.92 | 0.51 | 0.55 | 0.52 | 0.50 | 0.53 | 0.51 | 0.52 | 0.49 | 0.81 | 0.48 |
